# The hidden in plain sight: global, regional, and national trends in the pediatric burden of Klinefelter syndrome, 1990–2021

**DOI:** 10.3389/fgene.2025.1639699

**Published:** 2025-09-16

**Authors:** Guoqian Ma, Yuan Li, Fan Jia

**Affiliations:** ^1^ Department of Cardiology/Key Laboratory of Cardiovascular Disease of Yunnan Province/Clinical Medicine Center for Cardiovascular Disease of Yunnan Province, Yan’an Affiliated Hospital of Kunming Medical University, Kunming, China; ^2^ Ultrasound Department, Kunming Maternal and Child Health Hospital, Kunming, China

**Keywords:** Klinefelter syndrome (KS), children, public health, prevalence, DALYs, disability-adjusted life years

## Abstract

**Background:**

Klinefelter syndrome (KS) is the most common sex chromosome aneuploidy in males, but its epidemiology in children and adolescents remains poorly characterized worldwide. This study provides the first comprehensive global, regional, and national assessment of KS prevalence and disability-adjusted life years (DALYs) in individuals under 20 years from 1990 to 2021.

**Methods:**

We extracted data on KS prevalence and DALYs for individuals under 20 years of age from the Global Burden of Disease (GBD) 2021 database, covering 204 countries and territories. We evaluated temporal trends using the estimated annual percentage change (EAPC), stratified by age group, geographic region, and sociodemographic index (SDI) level.

**Findings:**

Between 1990 and 2021, the global number of KS cases in children and adolescents increased from 589,674 (95% UI, 440,342–770,284) to 690,885 (518,462–899,583), a 17.2% rise, while the overall prevalence rate per 100,000 remained stable (26.1 in 1990 to 26.2 in 2021). The global DALY burden attributed to KS rose by 20% over three decades, with marked disparities across SDI levels: in 2021, prevalence rates ranged from 17.1 per 100,000 (low-SDI) to 32.5 per 100,000 (high-SDI), and DALY rates varied from 0.05 to 0.15 per 100,000 across regions. High-SDI countries reported higher prevalence and DALY rates, likely reflecting superior diagnostic capacity and access to genetic services. In contrast, most low- and middle-SDI regions showed minimal changes in prevalence rates, despite increases in absolute case numbers, suggesting persistent underdiagnosis. Notably, children under 1 year of age and adolescents aged 15–19 represented the groups with the highest (49.5 per 100,000) and lowest (17.6 per 100,000) prevalence, respectively.

**Interpretation:**

KS continues to represent a largely undetected pediatric health burden, especially in low- and middle-SDI settings. The findings highlight the urgent need for enhanced awareness, early detection strategies, and equitable access to genetic services in global child health policy. Timely diagnosis and intervention can help prevent long-term developmental and health-related consequences of KS.

## Introduction

Klinefelter syndrome (KS) is a common yet under-recognised genetic disorder in males, defined by the presence of an extra X chromosome (most often a 47, XXY karyotype) (Zitzmann et al., 2021; Satoshi et al., 2020). It is the most frequent sex chromosome aneuploidy in men, with an estimated occurrence in approximately 1 in 500 to 1 in 1,000 male births worldwide (Ghisa et al., 2022). This high prevalence makes KS one of the most common chromosomal conditions seen in newborns across all populations. Early in life, the syndrome’s effects are often subtle—for example, mild speech delays, subtle hypotonia, or minor motor coordination difficulties may be present but easily overlooked (García-Payá et al., 2023). Classic clinical features include primary testicular failure resulting in hypergonadotropic hypogonadism (leading to androgen deficiency and infertility), disproportionately tall stature, and a spectrum of neurodevelopmental differences (Lucas-Herald et al., 2025). Affected boys frequently exhibit mild delays in speech and language development, learning disabilities, or social and behavioral challenges (Ridder et al., 2023). Notably, infants with KS usually appear normal at birth (with typical male genitalia), and obvious dysmorphic signs are absent in early childhood (Xu et al., 2022). This phenotypic normalcy in neonates, combined with the variable and often mild nature of early symptoms, means that KS can evade clinical detection in childhood.

Despite its relative frequency, KS remains profoundly underdiagnosed in pediatric and adolescent populations. The majority of affected individuals are not identified until adulthood, often incidentally during evaluations for delayed puberty or infertility (Groth et al., 2013). The mean age of diagnosis is in the mid-30s, reflecting the routine missed opportunities for earlier recognition (Maiburg et al., 2012). Estimates suggest that only about 25% of individuals with KS are ever formally diagnosed in their lifetime (Gravholt et al., 2018; Liang et al., 2022; Salonia et al., 2019; Aksglaede et al., 2013), and fewer than 10% of KS cases are detected before puberty (Wosnitzer and Paduch, 2013). In other words, over half to three-quarters of those born with KS are never aware of their condition during childhood or adolescence. This delay in diagnosis has important clinical implications: untreated KS in adolescence can lead to preventable consequences such as osteoporosis, metabolic syndrome, and psychological distress later in life (Kanakis et al., 2018). Indeed, research indicates that early diagnosis and timely interventions (including endocrine therapy and educational support) are associated with reduced comorbidity and improved long-term outcomes (Flannigan et al., 2018). Unfortunately, because many clinicians are unfamiliar with the variable pediatric presentation, a large number of KS cases in youth go unrecognized. KS is not typically associated with early mortality, but it does contribute to lifelong morbidity, including hormonal, metabolic, cardiovascular, and psychosocial effects (Mameli et al., 2022; Salzano et al., 2018), that can be quantified in terms of disability-adjusted life years (DALYs). However, because mortality is low in KS, traditional DALY metrics—heavily weighted by premature death—may underestimate the neurocognitive and psychosocial disability experienced by affected individuals. The global impact of KS in children remains poorly documented, and no prior study has provided a consolidated international overview of its prevalence and DALY burden in the pediatric age group. This paucity of data represents a critical knowledge gap: without robust epidemiological evidence, it is challenging to raise awareness, allocate resources, or develop early detection programs for KS at the global level.

In this study, we address the existing knowledge gaps by providing the first comprehensive global, regional, and national estimates of the prevalence of KS and its associated DALYs in individuals under 20 years of age from 1990 to 2021. Using data from the GBD 2021 analysis, we quantify the burden of KS across 204 countries and territories, highlighting variations by geography and over time. The objective of this work is to illuminate the epidemiological profile of KS in childhood and adolescence worldwide, thereby underscoring its relevance as a global health issue and laying the groundwork for improved early detection and management efforts.

## Methods

### Overview and methodological details

This cross-sectional study was approved by the Ethics Committee of Kunming yan’an Hospital, which waived the requirement for informed consent, as this research involved only data analysis without identifiable personal information. Data on prevalence and standardized disease definitions for KS in children aged 0–19 years were retrieved using the Global Health Data Exchange query tool developed by GBD collaborators. The GBD database is widely recognized as one of the most comprehensive and systematic global epidemiological frameworks, spearheaded by the Institute for Health Metrics and Evaluation (IHME) at the University of Washington (GBD, 2021 Causes of Death Collaborators et al., 2024). The primary aim of the GBD initiative is to quantify health losses attributable to various diseases, injuries, and risk factors globally.

The GBD approach facilitates comparative evaluations of prevalence and DALYs across countries, regions, and globally. Within this framework, disease burden is quantified through two principal metrics: prevalence and DALYs. DALYs represent the cumulative measure of health loss, calculated as the sum of Years of Life Lost (YLL) due to premature death and Years Lived with Disability (YLD). The respective formulas applied are:

YLL=Number of deaths×Standard life expectancy at the age of death

YLD=Prevalence of the condition×Disability weight

Disability weights, derived from expert consensus, range from 0 (perfect health) to 1 (equivalent to death). This robust methodological approach enables a scientifically rigorous understanding of global disease and injury burdens (GBD, 2021 Causes of Death Collaborators et al., 2024). However, these weights are not specific to pediatric KS; rather, they are adapted from generic health states (such as developmental delay, learning disability, hypogonadism, or infertility) that are considered analogous to the sequelae of KS. This methodological choice may underestimate or misclassify the unique disability burden of KS in children and adolescents. While DALYs provide a robust comparative metric, they may underestimate certain aspects of chronic disability and quality-of-life impairment relevant to KS.

The prevalence and DALYs of KS were estimated using the GBD standard tool DisMod-MR 2.1 (a Bayesian meta-regression model). This model integrates global epidemiological data and adjusts for diagnostic bias through covariates such as the Socio-demographic Index (SDI) and Healthcare Access and Quality (HAQ) Index. For regions with sparse data (e.g., low-SDI countries), spatiotemporal extrapolation was employed to generate estimates, while Markov chain Monte Carlo (MCMC) sampling was used to quantify uncertainty (reported as 95% uncertainty intervals [UIs]). Model validation adhered to the GBD cross-validation protocol (GBD, 2019 Diseases and Injuries Collaborators, 2020).

Data on KS prevalence and DALYs among children aged 0–19 years from 1990 to 2021 were extracted from the GBD database (https://vizhub.healthdata.org/gbd-results/; data accessed on 20 March 2025). Analyses incorporated multiple demographic dimensions, including sex, age groups (categorized as <1 year, 2–4 years, 5–9 years, 10–14 years, and 15–19 years), and geographical regions. However, analyses based on race or ethnicity were not performed due to the absence of relevant parameters in the GBD database. This study strictly adhered to the Strengthening the Reporting of Observational Studies in Epidemiology (STROBE) guidelines (von Elm et al., 2007).

### Sociodemographic index

The SDI is a composite measure reflecting the socioeconomic development level of a nation or region, encompassing economic structure and size, education level, standard of living, and social welfare and protection, among other factors (Li et al., 2024). The SDI ranges from 0 to 1, with higher scores indicating better socioeconomic status. The GBD classifies countries and regions into five categories based on SDI: low, low-middle, middle, high-middle, and high. This classification facilitates the examination of the influence of socioeconomic and geographical disparities on the burden of KS among children.

### Statistical analysis

Prevalence and DALYs per 100,000 population, along with 95% UIs, were calculated according to GBD statistical methods (GBD, 2019 Hearing Loss Collaborators, 2021). Joinpoint regression models were employed to determine annual percentage changes (APC) and associated 95% confidence intervals (CI), providing granular insights into annual trends within each distinct period (Lv et al., 2024). Additionally, log-transformed linear regression models calculated the estimated annual percentage change (EAPC) and corresponding 95% CI to evaluate temporal trends in prevalence and DALYs from 1990 to 2021. The EAPC is particularly valuable for assessing long-term trends, independent of short-term fluctuations (Zi et al., 2024). An EAPC value with a lower bound of the 95% CI above 0 indicates a rising trend, whereas an EAPC with an upper bound of the 95% CI below 0 indicates a declining trend. Curve-fitting analyses were conducted to investigate associations between disease burden indicators and SDI. All analyses were performed using R version 4.3.3, with statistical significance set at P < 0.05.

### Role of the funding source

Funders played no role in study design, data collection, analysis, interpretation, or manuscript preparation. All authors had unrestricted access to the study data and assumed full responsibility for the decision to submit the manuscript for publication.

## Result

### Global burden trends

#### Prevalence

**FIGURE 1:**
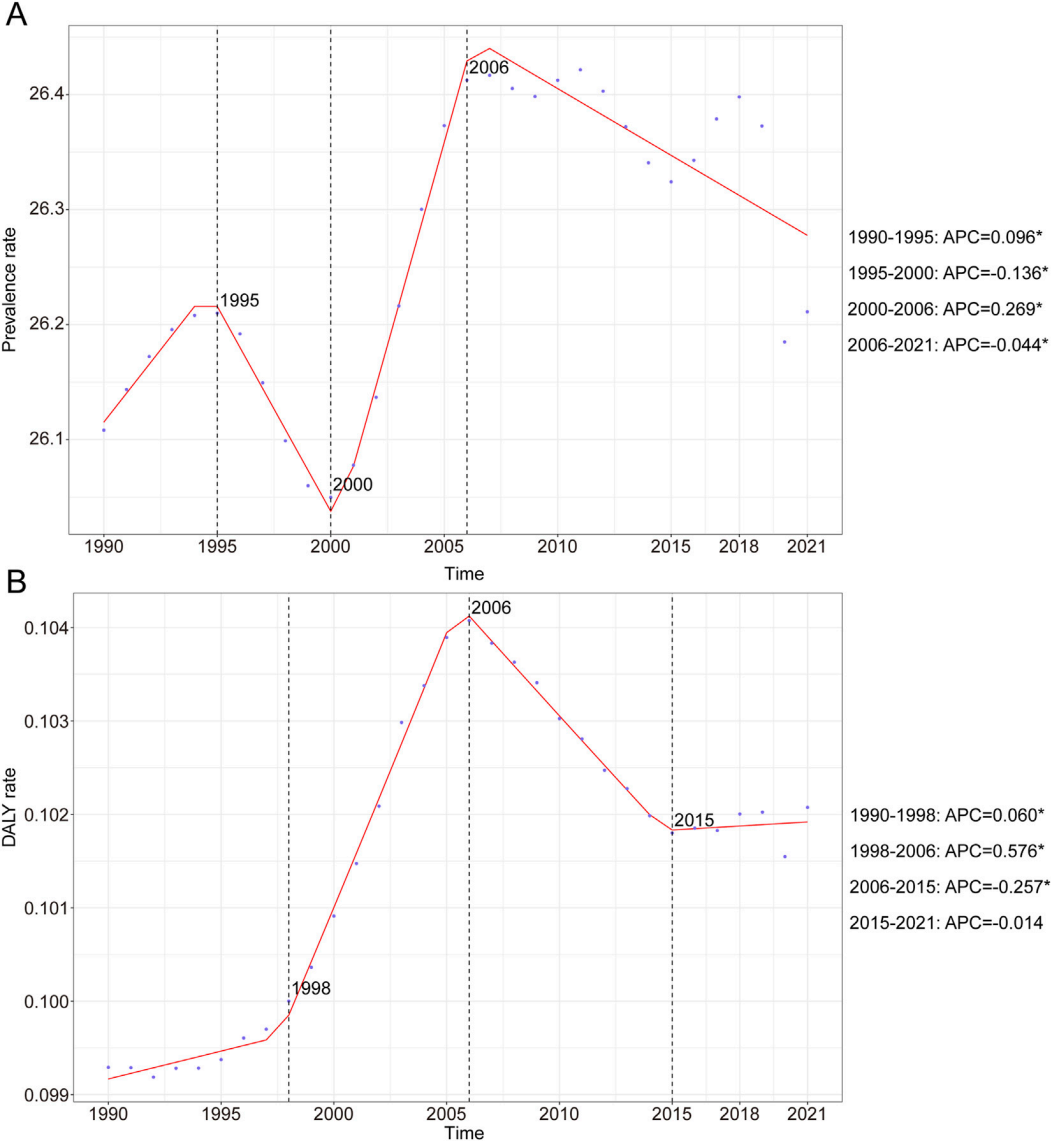
Annual percent change (APC) and trends in global childhood Klinefelter syndrome prevalence and disability-adjusted life years (DALYs) from 1990 to 2021. **(A)** Prevalence rate. **(B)** DALYs rate.

Data analysis revealed a dynamic global epidemiological trend for childhood KS, characterized by an “M-shaped” prevalence pattern. The highest APC occurred between 2000 and 2006, reaching 0.269% (95% CI, 0.200%–0.338%). Peak prevalence was recorded in 2006 at 26.34 cases per 100,000 (95% UI, 19.71–34.16). Conversely, the lowest APC was noted between 1995 and 2000, at −0.136% (95% CI, −0.205% to −0.067%), with the minimum prevalence observed in 2000 (26.05 per 100,000; 95% UI: 19.44–33.99) (Figure 1A). Between 1990 and 2021, the global prevalence of KS increased from 589,674 (95% UI, 440,342.03–770,284.36) to 690,884.60 cases (95% UI, 518,462.38–899,583.30), reflecting an overall growth of 17.16% (95% UI, 15.40–19.08). Prevalence rates during this period showed a modest increase from 26.11 (95% UI, 19.50–34.10) to 26.21 (95% UI, 19.67–34.13) per 100,000 individuals, representing an overall rise of 0.39% (95% UI, −1.12–2.04). The EAPC was 0.03 (95% CI, 0.02–0.05) (Table 1). Notably, prevalence rates of KS slightly increased across all age groups between 1990 and 2021, with increments ranging between 1% and 5% (Supplementary Figure S1A). In 2021, the highest prevalence occurred among children under 1 year of age, with 49.50 cases per 100,000 individuals (95% UI, 35.20–65.05). In contrast, adolescents aged 15–19 consistently exhibited the lowest prevalence, at 17.55 cases per 100,000 individuals (95% UI, 13.20–22.92) (Supplementary Figure S1A; Table 1).

**TABLE 1:** Prevalence of Klinefelter syndrome in children between 1990 and 2021 at the global and regional level.

Location	1990		2021		1990–2021		
	Prevalence cases	Prevalence rate	Prevalence cases	Prevalence rate	Cases change^a^	Rate change^a^	EAPC^b^
Global	589,674.00 (440,342.03,770,284.36)	26.11 (19.50,34.10)	690,884.60 (518,462.38,899,583.30)	26.21 (19.67,34.13)	17.16 (15.40,19.08)	0.39 (-1.12,2.04)	0.03 (0.02,0.05)
SDI							
High SDI	63,424.63 (47,929.39,82,409.91)	25.24 (19.07,32.79)	61,859.64 (46,965.94,80,224.15)	26.58 (20.18,34.47)	−2.47 (-4.54,-0.14)	5.33 (3.09,7.84)	0.10 (0.04,0.15)
High-middle SDI	84,022.61 (62,572.63,109,423.01)	22.70 (16.90,29.56)	68,385.04 (51,065.05,89,188.64)	22.54 (16.83,29.40)	−18.61 (-20.84,-16.32)	−0.69 (-3.41,2.11)	0.09 (0.01,0.16)
Middle SDI	170,980.86 (127,575.20,224,755.52)	22.36 (16.69,29.40)	167,987.15 (126,231.06,218,378.90)	22.42 (16.85,29.15)	−1.75 (-3.99,0.89)	0.26 (-2.02,2.96)	0.03 (0.01,0.04)
Low-middle SDI	175,676.04 (128,885.73,230,180.37)	29.72 (21.81,38.95)	208,889.77 (157,563.28,272,669.90)	27.33 (20.61,35.67)	18.91 (15.06,23.00)	−8.06 (-11.04,-4.89)	−0.27 (-0.29,-0.26)
Low SDI	95,126.33 (70,356.85,124,639.77)	34.03 (25.17,44.58)	183,278.56 (136,158.22,239,662.31)	31.37 (23.31,41.02)	92.67 (86.72,99.29)	−7.80 (-10.64,-4.63)	−0.28 (-0.30,-0.25)
Regions							
Andean Latin America	4,753.08 (3,502.03,6150.93)	25.07 (18.47,32.45)	5,322.14 (4,021.41,6872.86)	22.48 (16.99,29.03)	11.97 (3.09,22.87)	−10.34 (-17.45,-1.62)	−0.38 (-0.42,-0.35)
Australasia	1,093.24 (838.98,1424.93)	17.43 (13.37,22.71)	1,327.16 (994.69,1747.66)	17.60 (13.19,23.17)	21.40 (8.32,34.96)	0.98 (-9.90,12.26)	−0.11 (-0.24,0.03)
Caribbean	2,967.71 (2,224.84,3835.89)	19.66 (14.74,25.40)	3,098.68 (2,292.18,4096.89)	20.30 (15.02,26.84)	4.41 (-2.82,12.14)	3.29 (-3.86,10.94)	0.08 (0.04,0.11)
Central Asia	10,229.45 (7,611.70,13,193.55)	32.39 (24.10,41.78)	11,644.04 (8,774.26,15,228.95)	33.63 (25.34,43.98)	13.83 (7.89,20.88)	3.82 (-1.60,10.25)	0.21 (0.12,0.30)
Central Europe	7,702.26 (5,749.20,10,012.68)	19.61 (14.64,25.50)	4,918.30 (3,692.74,6419.38)	20.88 (15.68,27.25)	−36.14 (-38.61,-33.25)	6.44 (2.34,11.27)	0.26 (0.20,0.33)
Central Latin America	17,014.67 (12,779.33,22,344.47)	20.59 (15.47,27.04)	15,886.90 (11,955.81,20,905.87)	18.63 (14.02,24.51)	−6.63 (-10.55,-2.83)	−9.53 (-13.33,-5.85)	−0.32 (-0.34,-0.30)
Central Sub-Saharan Africa	10,824.54 (7,894.28,14,618.90)	34.93 (25.48,47.18)	23,834.33 (17,690.53,31,952.09)	32.40 (24.05,43.44)	120.19 (96.33,145.48)	−7.24 (-17.30,3.41)	−0.21 (-0.25,-0.18)
East Asia	81,386.92 (60,503.99,106,935.55)	17.69 (13.15,23.24)	65,139.03 (48,291.98,84,814.84)	18.88 (14.00,24.59)	−19.96 (-23.04,-16.71)	6.76 (2.66,11.10)	0.21 (0.13,0.28)
Eastern Europe	26,169.62 (19,746.99,33,634.51)	38.90 (29.35,50.00)	17,973.40 (13,380.96,23,499.18)	38.94 (28.99,50.91)	−31.32 (-33.67,-28.74)	0.10 (-3.32,3.85)	0.25 (0.14,0.37)
Eastern Sub-Saharan Africa	38,860.56 (28,374.81,50,865.66)	35.04 (25.59,45.87)	73,529.29 (55,013.02,95,765.15)	32.31 (24.17,42.08)	89.21 (82.43,97.44)	−7.80 (-11.11,-3.79)	−0.26 (-0.28,-0.25)
High-income Asia Pacific	9,045.88 (6,932.53,11,638.48)	17.97 (13.78,23.13)	5,793.28 (4,513.77,7497.94)	18.82 (14.66,24.35)	−35.96 (-38.84,-32.95)	4.68 (-0.03,9.60)	0.09 (-0.07,0.26)
High-income North America	23,616.51 (17,158.37,31,684.03)	28.90 (20.99,38.77)	24,822.51 (17,906.68,33,426.54)	27.72 (19.99,37.32)	5.11 (2.27,8.76)	−4.08 (-6.67,-0.75)	−0.31 (-0.40,-0.22)
North Africa and Middle East	47,693.48 (35,571.39,62,066.53)	26.98 (20.12,35.11)	59,413.74 (44,619.05,77,327.26)	25.12 (18.87,32.70)	24.57 (19.32,30.05)	−6.88 (-10.81,-2.79)	−0.17 (-0.19,-0.14)
Oceania	991.20 (735.95,1299.33)	29.44 (21.86,38.60)	1940.08 (1,409.80,2582.12)	30.38 (22.08,40.43)	95.73 (75.68,117.21)	3.17 (-7.40,14.50)	0.13 (0.12,0.15)
South Asia	168,923.95 (124,993.99,221,409.68)	31.14 (23.04,40.82)	189,171.18 (142,075.13,245,789.62)	27.68 (20.79,35.96)	11.99 (8.03,16.28)	−11.13 (-14.27,-7.72)	−0.42 (-0.44,-0.39)
Southeast Asia	53,458.70 (40,388.18,69,875.04)	24.31 (18.37,31.78)	53,240.16 (40,000.05,69,396.61)	23.22 (17.45,30.27)	−0.41 (-4.04,3.44)	−4.48 (-7.96,-0.78)	−0.11 (-0.13,-0.10)
Southern Latin America	2086.00 (1,555.42,2705.37)	10.76 (8.03,13.96)	1967.71 (1,476.77,2585.24)	10.09 (7.57,13.25)	−5.67 (-15.18,5.23)	−6.30 (-15.75,4.52)	−0.41 (-0.63,-0.19)
Southern Sub-Saharan Africa	8,776.22 (6,530.68,11,577.78)	33.17 (24.68,43.75)	10,146.05 (7,594.25,13,256.68)	32.45 (24.29,42.40)	15.61 (10.03,21.99)	−2.15 (-6.87,3.25)	−0.01 (-0.04,0.02)
Tropical Latin America	10,960.08 (7,983.67,14,711.65)	15.82 (11.53,21.24)	10,715.54 (7,845.56,14,239.73)	16.09 (11.78,21.38)	−2.23 (-6.35,2.03)	1.70 (-2.59,6.13)	0.06 (0.04,0.09)
Western Europe	28,800.15 (22,343.05,36,387.35)	29.28 (22.72,37.00)	29,231.51 (22,780.11,37,022.18)	31.87 (24.84,40.37)	1.50 (-2.33,5.28)	8.84 (4.73,12.90)	0.31 (0.26,0.36)
Western Sub-Saharan Africa	34,319.79 (25,440.62,44,871.67)	31.93 (23.67,41.74)	81,769.56 (60,648.12,107,278.14)	30.45 (22.58,39.94)	138.26 (131.07,146.06)	−4.64 (-7.52,-1.52)	−0.15 (-0.19,-0.11)

Abbreviations: EAPC, estimated annual percentage change; SDI, sociodemographic index; UI, uncertainty interval.

^a^ Change shows the percentage change.

^b^ EAPC, is expressed as 95% confidence interval.

#### DALYs

The KS-associated DALY rate exhibited an overall upward and then downward trend over the past three decades. The highest APC was recorded from 1998 to 2006 with a value of 0.576% (95% CI, 0.538%–0.614%) (Figure 1B). Furthermore, the peak DALYs were observed in 2006, with a DALY rate of 0.10 (95% UI, 0.05–0.21) per 100,000 people (Figure 1B). In 1990, the global DALYs attributed to KS were 2,242.60 (95% UI, 1,120.89–4,311.58), increasing to 2,690.53 (95% UI, 1,356.73–5,215.14) by 2021—an overall rise of 19.97% (95% UI, 15.67%–24.53%) (Table 2). The DALY rate rose marginally from 0.10 per 100,000 (95% UI, 0.05–0.19) in 1990 to 0.10 per 100,000 (95% UI, 0.05–0.20) in 2021, corresponding to a 2.80% increase (95% UI, −0.89%–6.71%). The EAPC was 0.11 (95% CI, 0.06–0.16) (Table 2). In 2021, children aged 15–19 years exhibited the highest KS-related DALY rate, at 0.18 per 100,000 (95% UI, 0.08–0.37), whereas the lowest rate was observed among children aged 10–14 years, at 0.06 per 100,000 (95% UI, 0.03–0.11) (Supplementary Figure S1B).

**TABLE 2:** DALYs of Klinefelter syndrome in children between 1990 and 2021 at the global and regional level.

Location	1990		2021		1990–2021		
	DALYs cases	DALYs rate	DALYs cases	DALYs rate	Cases change^a^	Rate change^a^	EAPC^b^
Global	2,242.60 (1,120.89,4311.58)	0.10 (0.05,0.19)	2,690.53 (1,356.73,5215.14)	0.10 (0.05,0.20)	19.97 (15.67,24.53)	2.80 (-0.89,6.71)	0.11 (0.06,0.16)
SDI region							
High SDI	270.91 (135.91,539.01)	0.11 (0.05,0.21)	266.05 (129.52,525.31)	0.11 (0.06,0.23)	−1.79 (-8.07,5.76)	6.05 (-0.72,14.21)	0.18 (0.12,0.25)
High-middle SDI	345.88 (170.66,714.43)	0.09 (0.05,0.19)	280.00 (137.55,551.28)	0.09 (0.05,0.18)	−19.05 (-25.10,-12.98)	−1.22 (-8.60,6.18)	0.02 (-0.07,0.12)
Middle SDI	665.88 (331.09,1291.50)	0.09 (0.04,0.17)	670.00 (331.56,1334.94)	0.09 (0.04,0.18)	0.62 (-4.92,5.83)	2.68 (-2.97,8.00)	0.14 (0.07,0.20)
Low-middle SDI	630.01 (312.52,1218.53)	0.11 (0.05,0.21)	806.70 (404.83,1582.50)	0.11 (0.05,0.21)	28.05 (17.43,40.44)	−0.99 (-9.20,8.58)	−0.04 (-0.07,-0.02)
Low SDI	328.19 (163.76,615.72)	0.12 (0.06,0.22)	665.89 (342.59,1286.29)	0.11 (0.06,0.22)	102.90 (86.41,122.65)	−2.90 (-10.79,6.55)	−0.13 (-0.15,-0.12)
Regions							
Andean Latin America	17.76 (8.60,34.98)	0.09 (0.05,0.18)	20.78 (10.18,41.34)	0.09 (0.04,0.17)	17.04 (-6.83,51.79)	−6.28 (-25.40,21.54)	−0.18 (-0.21,-0.14)
Australasia	4.75 (2.19,9.60)	0.08 (0.03,0.15)	5.54 (2.66,10.78)	0.07 (0.04,0.14)	16.65 (-15.57,59.38)	−2.97 (-29.77,32.57)	−0.12 (-0.24,-0.00)
Caribbean	11.60 (5.57,22.93)	0.08 (0.04,0.15)	12.17 (5.90,23.79)	0.08 (0.04,0.16)	4.96 (-14.62,27.90)	3.83 (-15.53,26.52)	0.19 (0.16,0.22)
Central Asia	38.22 (18.78,77.05)	0.12 (0.06,0.24)	43.31 (21.34,85.64)	0.13 (0.06,0.25)	13.31 (-7.23,39.69)	3.35 (-15.38,27.41)	0.24 (0.14,0.35)
Central Europe	32.39 (15.51,64.38)	0.08 (0.04,0.16)	20.73 (10.25,43.49)	0.09 (0.04,0.18)	−36.00 (-43.54,-27.23)	6.68 (-5.89,21.31)	0.13 (0.06,0.21)
Central Latin America	64.28 (32.03,127.37)	0.08 (0.04,0.15)	64.65 (32.32,126.97)	0.08 (0.04,0.15)	0.57 (-9.22,11.88)	−2.56 (-12.04,8.40)	−0.07 (-0.10,-0.05)
Central Sub-Saharan Africa	37.62 (17.27,73.41)	0.12 (0.06,0.24)	85.45 (40.78,165.95)	0.12 (0.06,0.23)	127.14 (73.29,202.70)	−4.31 (-27.00,27.51)	−0.12 (-0.15,-0.09)
East Asia	339.41 (162.97,661.66)	0.07 (0.04,0.14)	259.52 (129.51,519.85)	0.08 (0.04,0.15)	−23.54 (-30.38,-16.85)	1.99 (-7.14,10.92)	0.21 (0.05,0.37)
Eastern Europe	104.05 (52.71,211.61)	0.15 (0.08,0.31)	71.93 (35.44,143.04)	0.16 (0.08,0.31)	−30.87 (-40.22,-21.17)	0.75 (-12.88,14.90)	0.01 (-0.16,0.18)
Eastern Sub-Saharan Africa	134.26 (66.02,251.90)	0.12 (0.06,0.23)	269.51 (133.09,526.41)	0.12 (0.06,0.23)	100.74 (80.23,122.89)	−2.18 (-12.18,8.61)	−0.11 (-0.13,-0.10)
High-income Asia Pacific	41.65 (19.71,84.03)	0.08 (0.04,0.17)	25.53 (12.47,50.85)	0.08 (0.04,0.17)	−38.71 (-47.60,-28.09)	0.19 (-14.35,17.55)	0.02 (-0.13,0.17)
High-income North America	97.56 (48.80,190.86)	0.12 (0.06,0.23)	106.81 (49.87,210.33)	0.12 (0.06,0.23)	9.48 (-1.37,20.31)	−0.09 (-9.99,9.79)	−0.10 (-0.18,-0.02)
North Africa and Middle East	176.95 (86.55,340.50)	0.10 (0.05,0.19)	230.05 (113.39,453.51)	0.10 (0.05,0.19)	30.01 (17.31,46.96)	−2.82 (-12.31,9.85)	−0.11 (-0.15,-0.07)
Oceania	3.59 (1.65,7.22)	0.11 (0.05,0.21)	7.07 (3.15,14.00)	0.11 (0.05,0.22)	97.06 (36.13,176.36)	3.87 (-28.24,45.68)	0.17 (0.14,0.20)
South Asia	607.64 (304.44,1185.89)	0.11 (0.06,0.22)	748.50 (377.26,1468.97)	0.11 (0.06,0.21)	23.18 (12.31,36.79)	−2.24 (-10.87,8.56)	−0.10 (-0.13,-0.07)
Southeast Asia	201.65 (102.27,382.17)	0.09 (0.05,0.17)	210.26 (104.93,405.89)	0.09 (0.05,0.18)	4.27 (-5.27,13.66)	0.01 (-9.14,9.01)	0.02 (-0.00,0.05)
Southern Latin America	8.38 (3.99,16.68)	0.04 (0.02,0.09)	8.32 (4.00,16.47)	0.04 (0.02,0.08)	−0.65 (-17.35,21.81)	−1.31 (-17.91,20.99)	−0.21 (-0.43,0.01)
Southern Sub-Saharan Africa	32.52 (16.53,62.21)	0.12 (0.06,0.24)	38.87 (19.23,75.04)	0.12 (0.06,0.24)	19.52 (0.63,36.64)	1.16 (-14.83,15.65)	0.04 (-0.01,0.10)
Tropical Latin America	41.94 (20.49,80.83)	0.06 (0.03,0.12)	42.49 (20.68,83.87)	0.06 (0.03,0.13)	1.31 (-9.61,15.51)	5.38 (-5.98,20.15)	0.19 (0.16,0.22)
Western Europe	127.92 (64.22,258.96)	0.13 (0.07,0.26)	126.90 (61.11,255.92)	0.14 (0.07,0.28)	−0.80 (-12.64,12.64)	6.38 (-6.33,20.79)	0.26 (0.20,0.32)
Western Sub-Saharan Africa	118.46 (58.50,226.10)	0.11 (0.05,0.21)	292.14 (145.17,559.56)	0.11 (0.05,0.21)	146.61 (124.82,168.73)	−1.30 (-10.02,7.55)	−0.08 (-0.12,-0.04)

Abbreviations: EAPC, estimated annual percentage change; SDI, sociodemographic index; UI, uncertainty interval.

^a^ Change shows the percentage change; DALYs, disability-adjusted life years.

^b^ EAPC, is expressed as 95% confidence interval.

#### Regional trends in childhood KS by SDI

**FIGURE 2:**
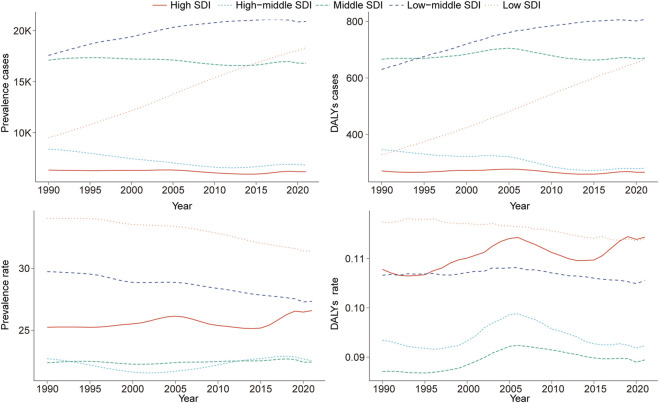
Epidemiologic trends in childhood Klinefelter syndrome prevalence and disability-adjusted life years (DALYs) rates across five Sociodemographic Index (SDI) areas from 1990 to 2021.

Compared with 1990, prevalence and DALY cases of childhood KS showed an upward trend in low and low-middle SDI regions by 2021. The largest increases were observed in the number of prevalent cases (92.67; 95% UI: 86.72–99.29) and DALYs (102.90; 95% UI: 86.41–122.65) (Tables 1, 2 Figure 2). Furthermore, the highest EAPC in childhood KS were observed in high SDI regions for both prevalence (EAPC = 0.10; 95% CI: 0.04–0.15) and DALYs (EAPC = 0.18; 95% CI: 0.12–0.25) (Tables 1, 2).

### Geographic regional trends in childhood KS

#### Prevalence

**FIGURE 3:**
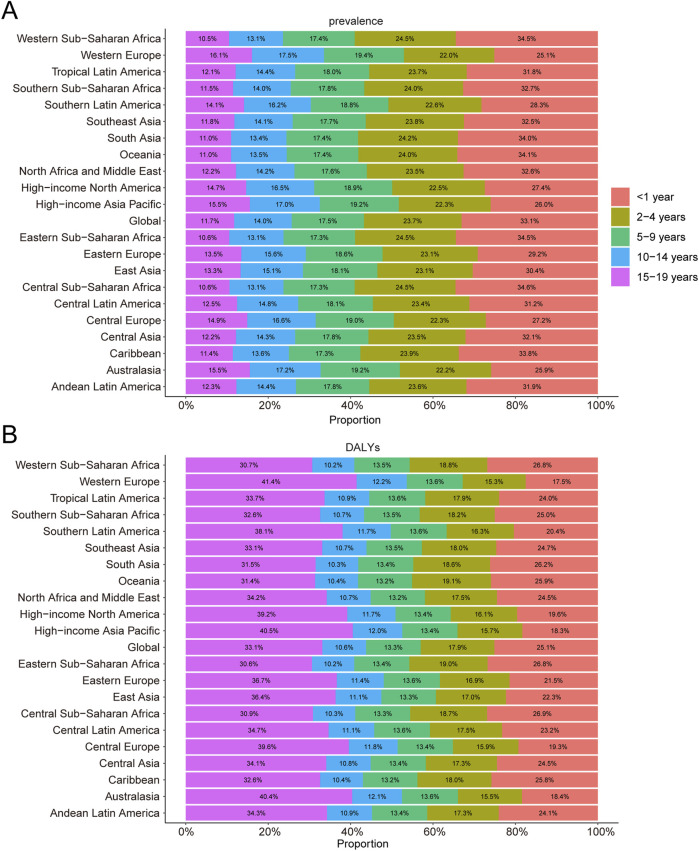
Age-specific percentages of childhood Klinefelter syndrome prevalence and disability-adjusted life years (DALYs) in 2021. **(A)** Prevalence. **(B)** DALYs.

In 2021, among 21 global regions, Central Sub-Saharan Africa exhibited the highest prevalence rate for KS among children younger than 1 year, accounting for 34.6% of all KS cases in this age group. In children aged 2–4 years, the highest prevalence was reported in Western, Central, and Eastern Sub-Saharan Africa, each region accounting for 24.5% of global cases. Western Europe reported the highest KS prevalence for children aged 5–9 years (19.4%), 10–14 years (17.5%), and 15–19 years (16.1%) (Figure 3A).

#### DALYs

In 2021, Central Sub-Saharan Africa had the highest DALY rate among children younger than 1 year, contributing 26.9% of the global DALYs from KS. Oceania recorded the highest DALY rate for children aged 2–4 years, accounting for 19.1% of the global DALYs in this age group. For children aged 5–9 years, DALY rates across the 21 regions ranged narrowly, contributing between 13.2% and 13.6% of global DALYs. Notably, Western Europe had the highest DALY rate for children aged 15–19 years, comprising 41.4% of the global DALYs in 2021 (Figure 3B).

### National trends in childhood KS

#### Prevalence

**FIGURE 4:**
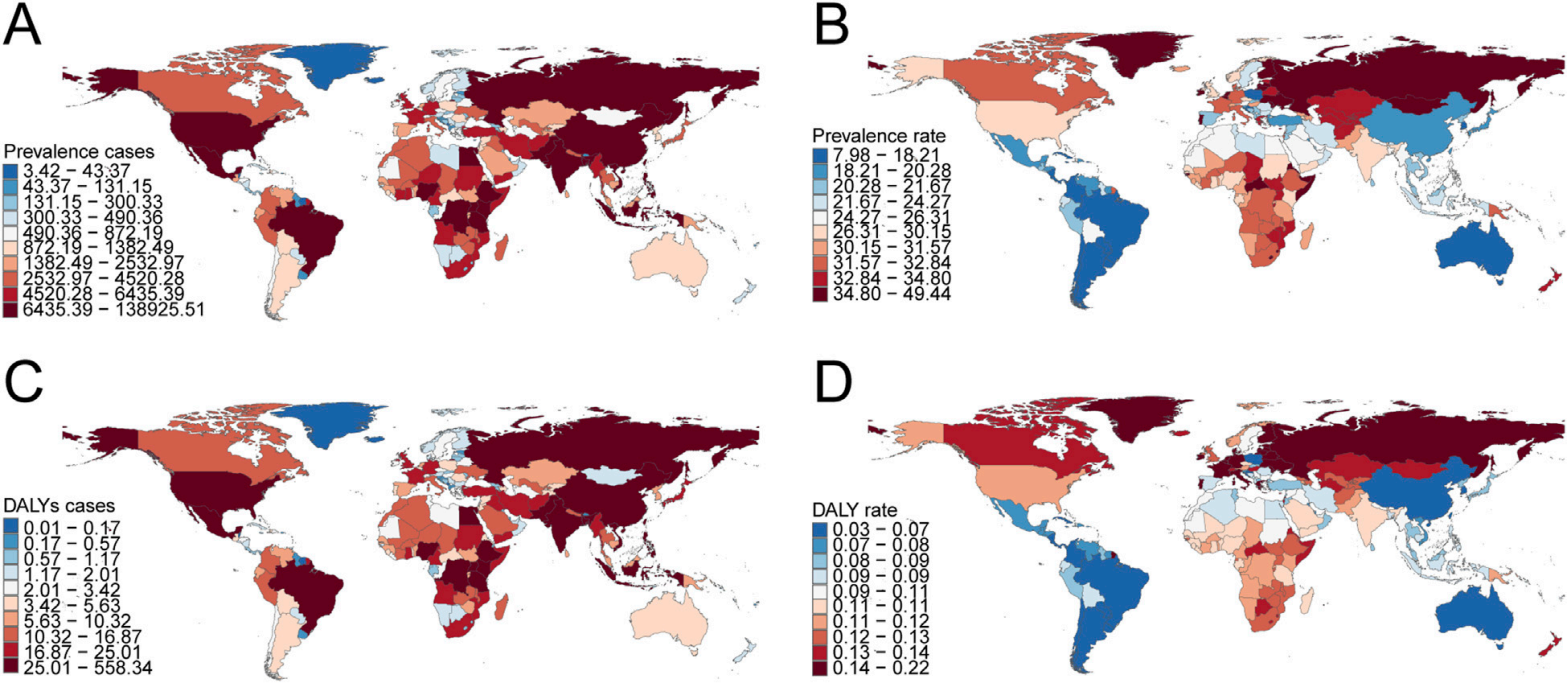
Prevalence and disability-adjusted life years (DALYs) of childhood Klinefelter syndrome across 204 countries and territories. **(A)** Number of prevalence cases. **(B)** Prevalence rate. **(C)** Number of DALYs. **(D)** DALY rate.

In 2021, India recorded the highest total number of childhood KS cases globally (137,550.01; 95% UI: 103,098.73–178,959.38), whereas Portugal exhibited the highest prevalence rate (48.95 per 100,000; 95% UI: 36.41–63.78). From 1990 to 2021, Spain demonstrated the largest increase in prevalence rates (EAPC = 1.51; 95% CI: 1.25–1.77), while Nepal had the largest decrease (EAPC = −0.66; 95% CI: −0.71 to −0.60) (Supplementary Table S1; Figures 4A,B). The global KS prevalence rate in 2021 was 26.21 per 100,000 (95% UI: 19.67–34.13), higher than the rates observed in 105 countries and lower than those in 99 countries out of 204 total nations.

#### DALYs

India had the highest global DALY count from KS in 2021 (552.81; 95% UI: 274.88–1,073.90), while Portugal had the highest DALY rate (0.22 per 100,000; 95% UI: 0.09–0.46) (Supplementary Table S2; Figures 4C,D). Between 1990 and 2021, Croatia experienced the greatest decline in DALYs (EAPC = −0.39; 95% CI: −0.64 to −0.14), whereas Spain showed the most significant increase (EAPC = 1.05; 95% CI: 0.87–1.23) (Supplementary Table S2). Globally, the DALY rate from KS in 2021 was 0.10 per 100,000 (95% UI: 0.05–0.20), with 98 countries exceeding this rate and 106 countries recording lower values.

## Discussion

### Principal findings and global health significance

This comprehensive analysis provides the first global estimate of KS burden in children and adolescents, revealing significant yet underappreciated impacts on population health. Previous study estimates a global birth prevalence of KS around 21.9 per 100,000 (approximately 0.022%) (Shen et al., 2024). This figure is markedly lower than the 0.1%–0.2% (1 in 500–1,000) prevalence suggested by neonatal cytogenetic surveys (Bojesen et al., 2003), underscoring that most KS cases remain unidentified in childhood. Such pervasive underdiagnosis means the true burden is substantially higher than reported, limiting our ability to address the needs of this population. Nevertheless, the cases that are captured in this study already amount to a considerable global pediatric burden of chronic health issues. Although KS contributed relatively low DALY rates (well under 1 per 100,000 globally), its importance lies in life-long impacts on quality of life rather than acute mortality. Children with KS often face neurodevelopmental challenges – language delays, learning disabilities, and psychosocial difficulties – that can hinder educational attainment and social integration (Boks et al., 2007). Without early recognition and support, these challenges can translate into significant cumulative disability. Thus, the finding that the recorded pediatric prevalence and DALY burden of KS have remained low globally should not be misconstrued as indicating that KS is negligible. Rather, this pattern likely reflects persistent under-detection and the historical neglect of this common chromosomal disorder in global health assessments. In a broader context, our results bring KS into the spotlight as a global health concern – albeit one that has been “hidden” in plain sight – affecting an estimated 1 in 600 males worldwide. By quantifying its prevalence and health loss among those under 20, this study fills a critical gap in the epidemiology of pediatric genetic disorders and establishes a baseline for tracking improvements in early diagnosis and care.

In this study, the “M-shaped” curve from 2000 to 2006 may partly reflect enhanced cytogenetic reporting, the adoption of new newborn screening protocols, and increased awareness of KS in high-resource settings. Improved diagnostic capabilities, such as more widespread karyotyping, contributed to transient spikes in case ascertainment. Notably, the “rise then fall” DALY trend was not uniform across SDI levels; regional analyses show this pattern was most pronounced in high-SDI and some upper-middle SDI regions, likely reflecting improved detection followed by stabilization as diagnostic practices plateaued. In low-SDI settings, DALY rates increased gradually, potentially due to persistent underdiagnosis and limited care access. The highest EAPCs for prevalence (0.10; 95% CI: 0.04–0.15) and DALYs (0.18; 95% CI: 0.12–0.25) occurred in high-SDI regions (Tables 1, 2), likely attributable to greater healthcare resources, comprehensive screening, and heightened clinical awareness enabling higher detection. Conversely, underdiagnosis in low-SDI regions may stem from limited karyotyping access, reduced clinician awareness, and sociocultural stigma. Strategies to enhance detection include expanded newborn screening, professional education, and improved registry infrastructure.

### Interpretation in context of health systems and pediatric genetics

When viewed alongside the literature, our findings reinforce a crucial point: the global health significance of KS lies not in early mortality or dramatic disability, but in its chronic, often unaddressed effects on health and wellbeing across the lifespan. Prior research has established that men with KS face elevated risks of numerous comorbidities, including metabolic syndrome (Zitzmann et al., 2021), type 2 diabetes (Gravholt et al., 2011), thromboembolism (Liang et al., 2022), osteoporosis (Willems et al., 2023), autoimmune disorders (Lucas-Herald et al., 2025), and certain malignancies (Win et al., 2020), contributing to a reduction in life expectancy by an estimated 2–6 years (Bojesen et al., 2011). These risks are already germinating in adolescence. For instance, adolescents with KS have higher rates of obesity and insulin resistance than their peers, as well as a propensity for psychiatric conditions such as anxiety and depression (Bojesen et al., 2010). The Million Veteran Program study in the US recently demonstrated that even men carrying an extra X or Y chromosome who were never clinically diagnosed showed significantly greater odds of cardiometabolic disease and neuropsychiatric issues compared to controls (Davis, 2024a). Notably, that large cohort genetic screening found a combined prevalence of X/Y aneuploidy around 1 in 370 men (0.27%) – higher than traditional newborn estimates – yet very few of those individuals had a documented KS diagnosis in medical records (Davis et al., 2024b). They were essentially “hidden” patients suffering the consequences without tailored care. This observation aligns with findings from Danish registry studies demonstrating that KS confers an increased standardized mortality risk, predominantly attributable to these comorbid conditions (Cho et al., 2020). Our study, focusing on those under 20, did not directly measure long-term outcomes like mortality; however, it quantifies the precursor state: the number of youths who, if left undiagnosed, are likely to fall through the cracks until perhaps presenting with infertility or severe disease decades later. In that sense, our work complements and extends prior studies by illuminating the *pediatric* segment of the KS population – a segment that previous global burden assessments had essentially omitted (Bai et al., 2024). By doing so, we contextualize KS within health systems: as a prevalent condition where the failure to integrate genetic diagnosis into primary care and pediatrics has led to preventable morbidity. The disparities we observed can be viewed as a barometer of health system capacity for genetic disorders. High detection rates in places like Northern Europe (e.g., Denmark, which achieved near-complete ascertainment of KS through past newborn studies (Herlihy et al., 2011)) show what is possible when proactive screening is in place. On the other hand, the near-absence of diagnosed cases in many low-income countries reflects systemic barriers – lack of trained specialists, limited access to karyotyping or chromosomal microarray tests, and low awareness among frontline providers (Wojcik et al., 2023). These deficiencies are not unique to KS; they apply to many rare pediatric conditions. Yet KS is among the most common congenital disorders (far more common than, for example, cystic fibrosis or phenylketonuria, which do receive newborn screening in many locales). Thus, the neglect of KS detection speaks to a broader policy blind spot in pediatric genetics and endocrinology. Our findings serve as a call to action to integrate genomic medicine equity into child health frameworks: every country should be capable of diagnosing a condition as frequent as KS. Closing these gaps is feasible – KS diagnosis requires a relatively simple chromosome analysis – but it demands political will and investment to ensure such tests and specialist consultations are accessible beyond tertiary centers in wealthy cities.

### Implications for public health and policy

#### Awareness

Raising awareness is fundamental to improving early detection of KS. Healthcare providers, including pediatricians, family doctors, school health professionals, and child development specialists, should receive targeted education regarding the subtle and variable presentation of KS in childhood. Key signs such as tall stature, disproportionate limb length, language delays, mild hypotonia, or behavioral/social challenges should prompt consideration of KS, even in the absence of obvious dysmorphic features. For infants, cryptorchidism or micropenis are straightforward clinical cues, and professional societies now recommend karyotype analysis in these scenarios. Educational campaigns, clinical decision support tools, and integration of KS red flags into electronic health records could facilitate timely referrals.

In low-resource settings, public and professional awareness campaigns are especially important to overcome social stigma and misconceptions regarding genetic conditions.

#### Diagnosis

##### Early and accurate diagnosis is essential for timely intervention

Screening protocols: The European Academy of Andrology and other societies recommend karyotype testing in boys with bilateral cryptorchidism, micropenis, or otherwise unexplained developmental delays. For adolescents, screening for KS should be considered in cases of pubertal delay, persistent small testes, or incomplete virilization.

Universal or targeted newborn screening: Universal newborn screening for KS remains controversial, primarily due to debates over cost-effectiveness and ethical considerations. In high-resource settings, pilot programs evaluating the addition of sex chromosome aneuploidy screening to routine newborn blood spots have shown promise, but widespread adoption is limited by cost and counseling infrastructure.

Feasibility in low-resource countries: Targeted screening of high-yield groups (e.g., males with infertility, adolescents with suggestive phenotypes, or children presenting to endocrinology/urology clinics) may be more practical than universal approaches in low- and middle-income countries. Development and scale-up of affordable molecular tests (such as PCR-based detection) can supplement traditional cytogenetic labs, particularly where karyotyping capacity is limited. International partnerships, donor funding, and integration with birth defect surveillance initiatives could further strengthen diagnostic infrastructure.

### Early intervention

Timely, multidisciplinary intervention can substantially improve the long-term outcomes of individuals with KS.

Endocrine management: Early androgen therapy (for example, low-dose oxandrolone) has shown benefit for muscle tone, bone health, and selected neurocognitive domains.

Developmental support: Early referral to speech, occupational, and physical therapy can address developmental delays, supporting academic and social success.

Long-term care: Establishment of multidisciplinary KS clinics—combining endocrinology, psychology, genetics, and education specialists—should be prioritized. In low-resource regions, telemedicine or periodic outreach clinics may help bridge the gap in specialist access.

### Cost-effectiveness and policy debate

Universal newborn screening for KS is not yet standard practice globally, due in part to the variable natural history, potential psychosocial harms, and resource constraints. While feasibility studies support the technical implementation of screening, rigorous cost-benefit analyses and outcome studies are needed before broad adoption (Tassone, 2014). Short-term pilot programs in high-prevalence settings, coupled with strong genetic counseling, may offer a pragmatic step forward.

### Ethical considerations

Prenatal and early diagnosis of KS, particularly via non-invasive prenatal testing (NIPT), raises important ethical questions. Parents must receive balanced counseling about the typically mild, manageable nature of KS, and the broad range of possible phenotypes. Avoiding unnecessary pregnancy termination due to misperceptions or stigma is an ethical imperative. In cases of prenatal or neonatal diagnosis, early linkage to multidisciplinary care is recommended to maximize developmental outcomes.

In conclusion, this study presents a novel global perspective on KS in children and adolescents, revealing a condition of significant prevalence that remains largely under-recognized.

### Limitations

This study has several important limitations. First, the analysis relies heavily on data from the GBD database, whose accuracy is constrained by the availability and quality of national registry data, a substantial number of undiagnosed pediatric KS cases, and the lack of detailed information on risk factors specific to childhood KS. Second, due to the clinical complexity of KS, missed diagnoses and misdiagnoses contribute significantly to underreporting, particularly in developing countries where diagnostic capabilities and awareness may be limited. Third, the GBD study’s estimates of the prevalence and DALYs for KS rely heavily on statistical modeling, particularly in countries with low SDI. Although the models adjust for diagnostic bias through covariates (e.g., SDI and healthcare accessibility), the uncertainty of estimates in regions lacking empirical data is substantially amplified.

## Conclusion

Childhood KS has shown an upward global trend in prevalence and associated disability burden over the past three decades. However, considerable regional disparities persist, underscoring the necessity for tailored, region-specific strategies in disease prevention, early diagnosis, and management. Future public health interventions should prioritize high-burden areas, particularly in lower SDI regions and countries with substantial case numbers, to effectively mitigate the growing global impact of KS.

## Data Availability

The original contributions presented in the study are included in the article/Supplementary Material, further inquiries can be directed to the corresponding author.

## Supplementary material

SUPPLEMENTARY FIGURE S1 Trends in prevalence and disability-adjusted life years (DALYs) of childhood Klinefelter syndrome by age. **(A)**, Prevalence rate. **(B)**, DALY rate.
