# Supplementary material for: The hidden in plain sight: global, regional, and national trends in the pediatric burden of Klinefelter syndrome, 1990–2021
Source: Front Genet. 2025 Sep 16;16:1639699. doi: 10.3389/fgene.2025.1639699 (PMC12479305; doi:10.3389/fgene.2025.1639699)
Supplement: Supplementary file 1 [file Table1.docx]

Table S1. prevalence of Klinefelter syndrome in children between 1990 and 2021 at the national levels.

| location | 1990 | |  | 2021 | |  | 1990-2021 | | |
| --- | --- | --- | --- | --- | --- | --- | --- | --- | --- |
|  | Prevalent cases | Prevalence rate |  | Prevalent cases | Prevalence rate |  | Cases change | Rate change | EAPC |
| Afghanistan | 1927.05(1386.49,2541.71) | 34.58(24.88,45.61) |  | 5849.88(4314.52,7791.08) | 32.91(24.27,43.83) |  | 203.57(165.54,245.47) | -4.83(-16.75,8.30) | -0.37(-0.48,-0.26) |
| Albania | 311.49(233.18,418.33) | 21.54(16.13,28.93) |  | 127.32(94.18,170.83) | 20.63(15.26,27.68) |  | -59.12(-64.18,-53.50) | -4.23(-16.07,8.94) | -0.29(-0.44,-0.14) |
| Algeria | 3538.51(2625.44,4641.65) | 26.18(19.42,34.34) |  | 4082.87(3030.25,5439.83) | 24.82(18.42,33.07) |  | 15.38(0.10,31.94) | -5.19(-17.74,8.42) | -0.07(-0.15,0.01) |
| American Samoa | 5.97(4.30,7.87) | 24.95(17.95,32.91) |  | 4.34(3.19,5.85) | 22.67(16.68,30.60) |  | -27.34(-36.45,-16.14) | -9.12(-20.50,4.90) | -0.27(-0.29,-0.24) |
| Andorra | 3.81(2.93,4.86) | 28.28(21.74,36.08) |  | 4.32(3.30,5.61) | 30.04(22.92,39.03) |  | 13.47(-0.28,27.93) | 6.22(-6.66,19.75) | 0.27(0.21,0.32) |
| Angola | 2053.55(1496.97,2793.64) | 35.61(25.96,48.45) |  | 6046.97(4389.57,8147.18) | 32.38(23.50,43.62) |  | 194.46(154.28,247.23) | -9.09(-21.49,7.20) | -0.27(-0.30,-0.24) |
| Antigua and Barbuda | 4.12(3.04,5.40) | 17.24(12.73,22.62) |  | 3.89(2.86,5.14) | 16.67(12.24,21.99) |  | -5.44(-17.47,8.35) | -3.31(-15.61,10.79) | -0.12(-0.14,-0.10) |
| Argentina | 1098.40(805.95,1457.00) | 8.43(6.19,11.18) |  | 1105.22(810.46,1468.69) | 8.06(5.91,10.71) |  | 0.62(-13.29,16.65) | -4.40(-17.62,10.83) | -0.32(-0.59,-0.04) |
| Armenia | 371.21(272.63,485.24) | 27.96(20.53,36.54) |  | 216.80(161.27,286.63) | 28.41(21.13,37.56) |  | -41.60(-49.60,-32.01) | 1.62(-12.30,18.31) | 0.24(0.11,0.37) |
| Australia | 745.57(567.45,975.90) | 14.42(10.97,18.87) |  | 888.47(664.80,1179.59) | 14.24(10.66,18.91) |  | 19.17(2.58,38.55) | -1.20(-14.95,14.87) | -0.21(-0.40,-0.02) |
| Austria | 447.74(345.95,580.20) | 23.90(18.47,30.97) |  | 459.40(347.94,601.04) | 26.18(19.83,34.25) |  | 2.60(-8.84,16.90) | 9.52(-2.70,24.78) | 0.33(0.27,0.38) |
| Azerbaijan | 963.34(709.13,1289.17) | 30.78(22.66,41.19) |  | 937.63(682.15,1228.56) | 30.77(22.39,40.32) |  | -2.67(-15.27,11.75) | -0.01(-12.95,14.81) | 0.12(0.02,0.22) |
| Bahamas | 21.28(15.58,28.57) | 19.76(14.48,26.54) |  | 21.64(16.07,28.86) | 18.77(13.95,25.04) |  | 1.69(-10.41,18.04) | -5.02(-16.32,10.25) | -0.21(-0.25,-0.17) |
| Bahrain | 47.56(35.98,63.30) | 23.77(17.98,31.64) |  | 89.11(67.30,117.69) | 22.08(16.68,29.17) |  | 87.35(65.11,111.19) | -7.10(-18.13,4.73) | -0.27(-0.33,-0.21) |
| Bangladesh | 17734.45(13113.52,23631.84) | 29.38(21.73,39.15) |  | 14838.76(10898.07,19655.36) | 24.33(17.87,32.23) |  | -16.33(-28.93,-3.99) | -17.19(-29.66,-4.98) | -0.61(-0.63,-0.59) |
| Barbados | 14.49(10.89,19.20) | 17.07(12.82,22.61) |  | 11.10(8.12,14.88) | 16.73(12.24,22.42) |  | -23.39(-33.84,-11.15) | -2.01(-15.37,13.65) | -0.09(-0.11,-0.07) |
| Belarus | 1096.79(822.37,1408.43) | 34.95(26.21,44.88) |  | 703.28(526.83,906.95) | 34.73(26.01,44.78) |  | -35.88(-44.53,-26.67) | -0.65(-14.06,13.62) | 0.19(0.09,0.28) |
| Belgium | 1093.33(825.33,1420.23) | 44.23(33.39,57.46) |  | 1187.15(928.29,1523.40) | 46.66(36.49,59.88) |  | 8.58(-5.01,22.93) | 5.49(-7.72,19.42) | 0.11(0.08,0.14) |
| Belize | 20.44(14.99,26.93) | 19.92(14.61,26.26) |  | 31.36(23.17,41.56) | 18.55(13.70,24.59) |  | 53.46(33.70,74.48) | -6.88(-18.87,5.87) | -0.27(-0.29,-0.25) |
| Benin | 949.08(701.51,1263.06) | 33.07(24.45,44.01) |  | 2266.44(1663.59,3004.49) | 30.15(22.13,39.97) |  | 138.80(106.18,175.93) | -8.83(-21.28,5.35) | -0.32(-0.34,-0.29) |
| Bermuda | 2.52(1.90,3.29) | 15.82(11.97,20.68) |  | 1.74(1.30,2.33) | 15.11(11.27,20.27) |  | -30.98(-39.76,-20.83) | -4.54(-16.68,9.50) | -0.12(-0.16,-0.09) |
| Bhutan | 94.10(69.21,125.09) | 27.63(20.32,36.73) |  | 61.05(43.89,81.87) | 23.94(17.21,32.11) |  | -35.12(-43.72,-24.71) | -13.33(-24.82,0.58) | -0.50(-0.53,-0.46) |
| Bolivia (Plurinational State of) | 921.94(684.65,1212.47) | 27.53(20.44,36.21) |  | 1111.65(829.87,1462.19) | 24.45(18.25,32.16) |  | 20.58(4.40,40.73) | -11.19(-23.11,3.65) | -0.41(-0.45,-0.37) |
| Bosnia and Herzegovina | 295.16(219.13,397.01) | 19.92(14.79,26.79) |  | 138.07(105.39,178.86) | 20.76(15.85,26.89) |  | -53.22(-59.64,-46.59) | 4.21(-10.09,19.00) | -0.03(-0.14,0.08) |
| Botswana | 256.59(189.92,345.74) | 34.65(25.65,46.69) |  | 301.13(220.64,403.03) | 32.84(24.06,43.95) |  | 17.36(1.84,35.24) | -5.23(-17.76,9.21) | -0.13(-0.15,-0.11) |
| Brazil | 10661.57(7765.30,14330.49) | 15.87(11.56,21.33) |  | 10344.72(7563.35,13768.49) | 16.18(11.83,21.54) |  | -2.97(-7.58,1.33) | 2.00(-2.85,6.52) | 0.07(0.04,0.10) |
| Brunei Darussalam | 23.02(17.09,30.84) | 20.01(14.85,26.81) |  | 25.74(18.96,33.91) | 19.93(14.68,26.26) |  | 11.80(-2.04,27.61) | -0.42(-12.74,13.66) | -0.14(-0.38,0.09) |
| Bulgaria | 504.38(375.22,671.86) | 21.34(15.88,28.43) |  | 304.97(231.29,407.40) | 23.56(17.87,31.48) |  | -39.54(-47.30,-30.95) | 10.40(-3.77,26.08) | 0.34(0.29,0.40) |
| Burkina Faso | 1938.19(1431.38,2596.01) | 34.26(25.30,45.89) |  | 4169.88(3045.68,5498.98) | 32.56(23.78,42.94) |  | 115.14(83.45,146.70) | -4.96(-18.96,8.99) | -0.20(-0.22,-0.18) |
| Burundi | 1163.22(829.35,1581.23) | 36.87(26.29,50.12) |  | 2510.86(1841.00,3300.26) | 34.52(25.31,45.37) |  | 115.85(85.20,147.91) | -6.38(-19.68,7.52) | -0.17(-0.22,-0.12) |
| Cabo Verde | 50.32(37.21,66.80) | 25.87(19.14,34.35) |  | 45.86(33.73,62.02) | 23.79(17.50,32.18) |  | -8.86(-19.89,3.87) | -8.04(-19.17,4.80) | -0.32(-0.35,-0.29) |
| Cambodia | 1616.55(1172.48,2144.25) | 28.39(20.59,37.66) |  | 1653.20(1186.85,2187.68) | 24.93(17.90,32.99) |  | 2.27(-11.37,16.38) | -12.19(-23.90,-0.08) | -0.45(-0.51,-0.39) |
| Cameroon | 1967.76(1456.47,2644.60) | 33.14(24.53,44.54) |  | 5190.78(3807.82,6875.24) | 30.71(22.53,40.68) |  | 163.79(125.64,206.79) | -7.34(-20.74,7.76) | -0.19(-0.22,-0.16) |
| Canada | 2428.49(1820.63,3160.09) | 31.68(23.75,41.23) |  | 2601.76(1963.54,3387.36) | 31.62(23.86,41.16) |  | 7.13(-6.36,23.67) | -0.21(-12.78,15.19) | 0.01(-0.01,0.04) |
| Central African Republic | 605.04(439.39,816.59) | 40.41(29.35,54.54) |  | 1126.69(807.89,1506.83) | 38.93(27.92,52.07) |  | 86.22(60.20,116.90) | -3.66(-17.13,12.21) | -0.13(-0.15,-0.11) |
| Chad | 1214.61(868.32,1631.91) | 34.45(24.63,46.29) |  | 3667.69(2683.07,4843.80) | 33.50(24.50,44.24) |  | 201.96(150.64,258.10) | -2.78(-19.30,15.29) | -0.13(-0.15,-0.11) |
| Chile | 862.28(650.59,1117.15) | 16.35(12.33,21.18) |  | 759.71(569.33,1017.62) | 15.52(11.63,20.79) |  | -11.90(-23.95,2.25) | -5.04(-18.03,10.21) | -0.32(-0.47,-0.18) |
| China | 78244.42(58086.92,102779.85) | 17.58(13.05,23.09) |  | 63043.89(46722.79,82275.97) | 18.86(13.98,24.61) |  | -19.43(-22.61,-15.88) | 7.26(3.02,11.98) | 0.23(0.15,0.30) |
| Colombia | 2994.61(2217.28,4072.32) | 19.89(14.73,27.05) |  | 2553.92(1896.65,3379.62) | 17.54(13.03,23.21) |  | -14.72(-25.34,-1.18) | -11.83(-22.81,2.17) | -0.48(-0.50,-0.45) |
| Comoros | 80.37(58.03,107.72) | 30.41(21.96,40.75) |  | 88.59(64.88,118.08) | 28.22(20.67,37.61) |  | 10.23(-2.95,27.24) | -7.20(-18.29,7.13) | -0.28(-0.31,-0.24) |
| Congo | 439.19(322.13,587.69) | 33.10(24.28,44.30) |  | 773.18(565.16,1043.58) | 31.09(22.73,41.96) |  | 76.05(51.41,102.80) | -6.08(-19.22,8.19) | -0.13(-0.19,-0.08) |
| Cook Islands | 1.90(1.42,2.55) | 22.12(16.51,29.57) |  | 1.02(0.76,1.34) | 19.70(14.66,25.79) |  | -46.40(-53.19,-38.49) | -10.95(-22.23,2.19) | -0.36(-0.38,-0.33) |
| Costa Rica | 239.05(179.12,314.91) | 16.86(12.63,22.21) |  | 213.41(158.08,280.18) | 15.59(11.55,20.47) |  | -10.73(-22.12,3.68) | -7.52(-19.33,7.41) | -0.28(-0.32,-0.23) |
| Croatia | 2357.20(1705.33,3176.99) | 34.07(24.65,45.92) |  | 4658.00(3397.38,6227.27) | 32.38(23.62,43.29) |  | -38.05(-46.24,-29.42) | 1.99(-11.50,16.19) | -0.12(-0.15,-0.08) |
| Cuba | 211.70(156.42,282.53) | 15.96(11.79,21.30) |  | 131.15(96.19,170.36) | 16.28(11.94,21.14) |  | -34.66(-43.24,-24.88) | -0.63(-13.68,14.25) | -0.38(-0.64,-0.12) |
| Cyprus | 570.03(414.48,750.46) | 15.61(11.35,20.55) |  | 372.44(279.72,498.46) | 15.51(11.65,20.76) |  | 13.16(0.69,29.75) | 2.83(-8.50,17.91) | -0.09(-0.12,-0.07) |
| Czechia | 83.80(62.87,112.10) | 32.35(24.27,43.27) |  | 94.83(71.58,124.79) | 33.26(25.11,43.77) |  | -20.10(-29.88,-8.71) | 9.52(-3.88,25.15) | 0.05(-0.01,0.12) |
| C么te d'Ivoire | 752.47(566.62,977.39) | 24.72(18.61,32.11) |  | 601.19(447.20,793.00) | 27.07(20.14,35.71) |  | 97.61(68.51,130.97) | -4.96(-18.95,11.09) | 0.52(0.38,0.65) |
| Democratic People's Republic of Korea | 1687.59(1247.52,2235.36) | 21.67(16.02,28.70) |  | 1368.21(1009.25,1805.98) | 20.72(15.29,27.35) |  | -18.93(-31.07,-5.59) | -4.35(-18.68,11.38) | -0.24(-0.29,-0.20) |
| Democratic Republic of the Congo | 7481.83(5460.36,10105.34) | 34.56(25.22,46.68) |  | 15414.87(11377.96,20558.27) | 32.18(23.75,42.91) |  | 106.03(76.10,141.27) | -6.89(-20.42,9.03) | -0.20(-0.24,-0.16) |
| Denmark | 382.78(284.05,503.23) | 30.62(22.72,40.25) |  | 413.46(313.63,540.57) | 31.95(24.23,41.77) |  | 8.01(-5.54,24.84) | 4.35(-8.74,20.60) | 0.07(0.03,0.11) |
| Djibouti | 70.75(52.87,94.96) | 31.55(23.57,42.34) |  | 167.46(125.82,226.67) | 31.69(23.81,42.90) |  | 136.69(102.61,175.24) | 0.47(-14.00,16.83) | 0.05(0.01,0.08) |
| Dominica | 6.51(4.78,8.71) | 19.87(14.59,26.59) |  | 3.57(2.61,4.70) | 18.38(13.43,24.19) |  | -45.18(-52.96,-37.77) | -7.52(-20.64,4.98) | -0.25(-0.30,-0.20) |
| Dominican Republic | 686.33(501.76,913.65) | 19.60(14.33,26.09) |  | 738.24(541.51,989.48) | 18.97(13.92,25.43) |  | 7.56(-7.91,26.77) | -3.18(-17.11,14.11) | -0.14(-0.19,-0.10) |
| Ecuador | 1295.36(954.59,1714.17) | 26.13(19.26,34.58) |  | 1527.03(1125.20,1969.30) | 23.08(17.01,29.76) |  | 17.88(4.24,35.32) | -11.67(-21.90,1.40) | -0.44(-0.48,-0.40) |
| Egypt | 7901.91(5789.08,10558.57) | 28.30(20.73,37.81) |  | 11740.55(8716.08,15429.10) | 25.22(18.72,33.15) |  | 48.58(28.34,72.04) | -10.88(-23.02,3.20) | -0.20(-0.27,-0.13) |
| El Salvador | 619.72(453.09,826.47) | 22.49(16.44,29.99) |  | 490.36(365.75,651.85) | 20.59(15.35,27.36) |  | -20.87(-30.96,-7.83) | -8.47(-20.14,6.62) | -0.36(-0.42,-0.30) |
| Equatorial Guinea | 85.86(63.02,113.89) | 35.78(26.26,47.46) |  | 238.07(175.59,319.00) | 30.91(22.80,41.42) |  | 177.27(138.88,220.95) | -13.60(-25.56,0.01) | -0.47(-0.50,-0.44) |
| Eritrea | 741.82(548.57,989.75) | 37.72(27.89,50.32) |  | 1139.98(837.51,1554.90) | 35.44(26.04,48.34) |  | 53.67(31.25,79.75) | -6.04(-19.75,9.91) | -0.22(-0.24,-0.20) |
| Estonia | 154.33(115.99,202.61) | 33.60(25.25,44.11) |  | 94.95(71.06,125.96) | 33.87(25.35,44.94) |  | -38.48(-46.48,-29.38) | 0.82(-12.31,15.72) | 0.14(0.07,0.22) |
| Eswatini | 172.38(126.31,239.53) | 36.13(26.47,50.20) |  | 186.58(137.84,250.60) | 34.87(25.76,46.84) |  | 8.24(-5.41,25.20) | -3.47(-15.65,11.66) | -0.06(-0.10,-0.03) |
| Ethiopia | 10916.80(8006.59,14680.76) | 37.05(27.17,49.82) |  | 18480.33(13597.87,24492.77) | 32.31(23.77,42.82) |  | 69.28(57.53,82.90) | -12.79(-18.84,-5.77) | -0.48(-0.50,-0.47) |
| Fiji | 86.11(63.19,114.54) | 24.01(17.62,31.93) |  | 83.27(59.33,111.08) | 23.75(16.92,31.68) |  | -3.30(-18.24,12.02) | -1.07(-16.36,14.60) | 0.04(0.02,0.07) |
| Finland | 492.87(370.82,646.43) | 38.85(29.23,50.95) |  | 446.92(338.83,595.98) | 38.91(29.50,51.89) |  | -9.32(-20.77,4.75) | 0.17(-12.48,15.71) | -0.07(-0.15,0.01) |
| France | 4106.48(3372.49,4968.96) | 25.44(20.89,30.78) |  | 5032.57(3925.95,6302.40) | 31.95(24.92,40.01) |  | 22.55(4.73,40.06) | 25.59(7.33,43.53) | 0.67(0.39,0.95) |
| Gabon | 159.07(114.10,215.55) | 31.30(22.45,42.42) |  | 234.56(171.83,310.55) | 28.36(20.78,37.55) |  | 47.46(27.25,71.59) | -9.40(-21.81,5.43) | -0.33(-0.36,-0.31) |
| Gambia | 174.23(128.96,233.33) | 31.01(22.95,41.53) |  | 371.59(271.15,492.03) | 29.34(21.41,38.85) |  | 113.28(85.26,141.21) | -5.40(-17.83,6.98) | -0.19(-0.21,-0.17) |
| Georgia | 505.04(378.03,663.73) | 28.11(21.04,36.94) |  | 288.17(211.38,386.48) | 31.01(22.75,41.59) |  | -42.94(-50.81,-34.25) | 10.33(-4.89,27.13) | 0.52(0.42,0.63) |
| Germany | 5428.69(3984.50,7035.47) | 31.32(22.99,40.60) |  | 5227.41(3961.52,6815.43) | 32.78(24.84,42.74) |  | -3.71(-16.91,8.50) | 4.65(-9.70,17.92) | 0.18(0.14,0.23) |
| Ghana | 2617.84(1886.31,3446.98) | 31.69(22.84,41.73) |  | 4814.25(3549.52,6344.60) | 29.51(21.76,38.89) |  | 83.90(57.03,113.29) | -6.89(-20.50,7.98) | -0.20(-0.23,-0.17) |
| Greece | 839.64(636.43,1079.29) | 29.87(22.64,38.40) |  | 629.87(473.14,821.44) | 33.06(24.83,43.11) |  | -24.98(-35.77,-15.14) | 10.66(-5.25,25.18) | 0.40(0.37,0.43) |
| Greenland | 8.28(6.14,11.11) | 46.30(34.31,62.10) |  | 6.72(5.04,8.74) | 43.70(32.82,56.88) |  | -18.92(-30.10,-7.83) | -5.62(-18.63,7.29) | -0.26(-0.31,-0.20) |
| Grenada | 8.10(5.94,10.89) | 19.21(14.08,25.84) |  | 5.56(4.11,7.31) | 18.48(13.64,24.28) |  | -31.29(-40.13,-20.38) | -3.78(-16.16,11.50) | -0.03(-0.11,0.05) |
| Guam | 11.93(8.87,16.19) | 22.03(16.38,29.89) |  | 10.61(7.86,13.77) | 21.89(16.21,28.41) |  | -11.03(-23.30,4.74) | -0.64(-14.35,16.96) | -0.05(-0.08,-0.01) |
| Guatemala | 1207.05(865.74,1582.69) | 24.58(17.63,32.23) |  | 1385.35(1002.87,1839.30) | 20.94(15.16,27.81) |  | 14.77(0.91,32.05) | -14.80(-25.09,-1.97) | -0.56(-0.60,-0.51) |
| Guinea | 1135.44(822.33,1502.80) | 34.54(25.02,45.72) |  | 2354.99(1711.06,3143.59) | 31.55(22.92,42.11) |  | 107.41(78.45,137.87) | -8.66(-21.42,4.75) | -0.26(-0.32,-0.21) |
| Guinea-Bissau | 217.55(158.67,290.86) | 36.99(26.98,49.46) |  | 395.96(283.51,515.39) | 35.40(25.35,46.08) |  | 82.01(58.58,117.20) | -4.30(-16.62,14.20) | -0.10(-0.14,-0.07) |
| Guyana | 88.52(65.29,117.13) | 23.20(17.11,30.69) |  | 62.77(45.74,84.94) | 22.36(16.29,30.26) |  | -29.09(-38.11,-18.96) | -3.61(-15.87,10.17) | -0.21(-0.25,-0.18) |
| Haiti | 862.63(626.80,1162.35) | 25.78(18.73,34.73) |  | 1379.22(1009.10,1818.02) | 24.59(17.99,32.42) |  | 59.89(38.27,84.62) | -4.59(-17.49,10.17) | -0.11(-0.17,-0.06) |
| Honduras | 591.15(435.49,800.91) | 21.78(16.05,29.51) |  | 855.92(630.26,1164.38) | 19.67(14.49,26.76) |  | 44.79(25.24,64.98) | -9.69(-21.88,2.90) | -0.35(-0.39,-0.32) |
| Hungary | 819.07(606.34,1082.22) | 28.22(20.89,37.29) |  | 583.41(441.48,770.10) | 31.11(23.54,41.06) |  | -28.77(-37.11,-17.37) | 10.23(-2.68,27.87) | 0.66(0.48,0.84) |
| Iceland | 24.65(18.59,32.22) | 29.17(22.00,38.12) |  | 26.96(20.52,35.42) | 30.26(23.03,39.76) |  | 9.38(-4.26,24.18) | 3.74(-9.19,17.78) | 0.11(0.09,0.13) |
| India | 127889.60(94761.38,167436.87) | 31.16(23.09,40.80) |  | 137550.01(103098.73,178959.38) | 27.48(20.60,35.75) |  | 7.55(3.16,12.18) | -11.82(-15.42,-8.02) | -0.44(-0.47,-0.41) |
| Indonesia | 21448.15(16167.44,27960.88) | 24.52(18.48,31.97) |  | 21359.74(15846.51,27799.07) | 23.68(17.56,30.81) |  | -0.41(-4.58,4.14) | -3.45(-7.50,0.96) | -0.06(-0.11,-0.02) |
| Iran (Islamic Republic of) | 8439.71(6341.71,10828.79) | 26.78(20.13,34.37) |  | 6258.59(4690.90,8078.55) | 24.21(18.14,31.25) |  | -25.84(-28.80,-22.99) | -9.62(-13.23,-6.15) | -0.13(-0.27,0.01) |
| Iraq | 2905.05(2160.00,3835.32) | 28.21(20.97,37.24) |  | 4325.86(3183.47,5759.79) | 24.53(18.05,32.66) |  | 48.91(28.91,74.77) | -13.05(-24.73,2.05) | -0.48(-0.53,-0.43) |
| Ireland | 566.39(424.76,726.15) | 42.62(31.97,54.65) |  | 591.62(441.05,778.09) | 44.77(33.37,58.87) |  | 4.45(-8.47,19.63) | 5.02(-7.97,20.28) | 0.12(0.08,0.16) |
| Israel | 619.37(471.47,817.68) | 30.87(23.50,40.76) |  | 1070.34(801.38,1406.98) | 31.73(23.76,41.71) |  | 72.81(53.05,94.62) | 2.78(-8.97,15.75) | 0.08(0.07,0.10) |
| Italy | 4098.56(3144.70,5233.74) | 30.09(23.09,38.43) |  | 3372.74(2590.63,4381.00) | 32.18(24.72,41.80) |  | -17.71(-20.84,-14.31) | 6.95(2.88,11.37) | 0.08(0.03,0.14) |
| Jamaica | 179.27(131.68,239.01) | 16.41(12.05,21.88) |  | 131.88(98.63,177.67) | 16.17(12.10,21.79) |  | -26.44(-36.49,-13.90) | -1.43(-14.90,15.36) | -0.10(-0.12,-0.08) |
| Japan | 6109.20(4739.48,7819.64) | 18.31(14.21,23.44) |  | 4226.12(3289.04,5385.44) | 19.90(15.49,25.36) |  | -30.82(-33.58,-28.36) | 8.70(4.37,12.57) | 0.26(0.12,0.41) |
| Jordan | 486.15(359.26,649.19) | 23.19(17.14,30.96) |  | 1006.33(738.63,1327.07) | 20.43(15.00,26.95) |  | 107.00(79.31,139.56) | -11.88(-23.67,1.98) | -0.41(-0.44,-0.38) |
| Kazakhstan | 2197.29(1620.51,2888.65) | 33.08(24.39,43.48) |  | 2290.17(1684.67,3054.07) | 34.06(25.05,45.42) |  | 4.23(-10.28,19.90) | 2.97(-11.36,18.46) | 0.22(0.14,0.30) |
| Kenya | 4329.40(3182.91,5653.46) | 31.39(23.08,40.99) |  | 7301.13(5443.40,9524.78) | 29.68(22.13,38.72) |  | 68.64(63.28,73.26) | -5.45(-8.46,-2.86) | -0.12(-0.16,-0.07) |
| Kiribati | 11.87(8.50,15.99) | 32.39(23.19,43.61) |  | 16.23(11.85,21.91) | 30.37(22.17,40.99) |  | 36.72(19.99,61.74) | -6.24(-17.71,10.92) | -0.18(-0.21,-0.15) |
| Kuwait | 139.72(103.94,185.48) | 20.37(15.15,27.04) |  | 213.90(162.20,277.52) | 19.53(14.81,25.33) |  | 53.10(30.74,74.07) | -4.13(-18.13,9.00) | -0.19(-0.22,-0.15) |
| Kyrgyzstan | 696.28(518.77,920.23) | 32.98(24.57,43.58) |  | 941.45(689.84,1243.38) | 33.35(24.44,44.05) |  | 35.21(18.96,54.39) | 1.13(-11.02,15.48) | 0.14(0.06,0.21) |
| Lao People's Democratic Republic | 663.12(477.83,894.69) | 29.23(21.06,39.44) |  | 782.48(574.11,1059.52) | 26.16(19.19,35.42) |  | 18.00(1.10,35.42) | -10.52(-23.33,2.69) | -0.46(-0.50,-0.41) |
| Latvia | 266.00(195.61,356.20) | 35.35(26.00,47.34) |  | 139.51(103.38,182.99) | 36.04(26.71,47.27) |  | -47.55(-55.11,-39.60) | 1.95(-12.75,17.40) | 0.29(0.16,0.41) |
| Lebanon | 333.41(247.00,443.79) | 24.90(18.44,33.14) |  | 371.31(277.13,494.15) | 22.28(16.63,29.66) |  | 11.37(-4.40,27.17) | -10.50(-23.17,2.20) | -0.35(-0.38,-0.33) |
| Lesotho | 316.50(227.98,435.14) | 37.80(27.23,51.97) |  | 299.54(218.81,400.61) | 35.71(26.09,47.76) |  | -5.36(-20.09,9.79) | -5.53(-20.24,9.59) | -0.12(-0.14,-0.11) |
| Liberia | 442.77(327.75,592.38) | 32.41(23.99,43.36) |  | 810.94(607.94,1059.22) | 29.07(21.79,37.96) |  | 83.15(58.47,113.89) | -10.32(-22.40,4.73) | -0.23(-0.27,-0.20) |
| Libya | 548.04(409.75,713.90) | 23.74(17.75,30.93) |  | 473.13(350.50,617.66) | 22.66(16.79,29.59) |  | -13.67(-24.49,-1.43) | -4.54(-16.50,8.99) | -0.07(-0.10,-0.03) |
| Lithuania | 383.13(276.54,513.72) | 34.59(24.97,46.38) |  | 191.37(141.93,252.35) | 35.72(26.49,47.10) |  | -50.05(-56.84,-41.96) | 3.26(-10.77,19.99) | 0.23(0.13,0.34) |
| Luxembourg | 28.32(21.20,37.37) | 32.10(24.03,42.36) |  | 44.70(33.42,58.05) | 33.13(24.77,43.02) |  | 57.82(38.03,80.95) | 3.21(-9.73,18.33) | 0.14(0.11,0.16) |
| Madagascar | 2196.93(1614.99,2900.22) | 32.66(24.01,43.12) |  | 4520.28(3259.76,6135.74) | 30.25(21.81,41.06) |  | 105.75(77.60,139.13) | -7.39(-20.07,7.63) | -0.23(-0.26,-0.21) |
| Malawi | 2055.08(1497.31,2742.77) | 36.87(26.87,49.21) |  | 3409.01(2534.93,4482.04) | 32.19(23.94,42.32) |  | 65.88(42.79,99.47) | -12.71(-24.86,4.97) | -0.45(-0.50,-0.41) |
| Malaysia | 1875.68(1395.93,2470.88) | 22.48(16.73,29.62) |  | 2138.11(1598.21,2849.41) | 20.85(15.58,27.78) |  | 13.99(-1.84,33.20) | -7.26(-20.14,8.36) | -0.31(-0.34,-0.28) |
| Maldives | 27.06(20.00,35.68) | 21.08(15.58,27.80) |  | 23.86(17.75,31.29) | 18.31(13.63,24.02) |  | -11.83(-24.00,1.50) | -13.16(-25.15,-0.03) | -0.42(-0.55,-0.30) |
| Mali | 1633.40(1191.89,2195.48) | 33.03(24.10,44.39) |  | 4399.40(3224.19,5866.62) | 30.91(22.65,41.22) |  | 169.34(130.92,217.50) | -6.41(-19.76,10.33) | -0.22(-0.23,-0.21) |
| Malta | 35.79(26.83,47.13) | 31.06(23.28,40.89) |  | 26.79(20.57,34.83) | 31.90(24.50,41.49) |  | -25.17(-34.47,-13.39) | 2.73(-10.04,18.89) | 0.10(0.04,0.17) |
| Marshall Islands | 7.59(5.40,10.22) | 28.34(20.17,38.17) |  | 6.17(4.49,8.41) | 26.74(19.45,36.47) |  | -18.70(-29.07,-6.28) | -5.63(-17.66,8.79) | -0.12(-0.16,-0.09) |
| Mauritania | 333.09(244.22,439.03) | 29.38(21.54,38.72) |  | 611.59(449.96,807.32) | 26.22(19.29,34.62) |  | 83.61(57.11,112.49) | -10.73(-23.61,3.31) | -0.33(-0.35,-0.30) |
| Mauritius | 90.05(66.46,117.75) | 20.90(15.43,27.33) |  | 59.43(43.32,78.09) | 20.02(14.59,26.30) |  | -34.01(-41.94,-24.21) | -4.24(-15.76,9.98) | -0.13(-0.16,-0.11) |
| Mexico | 8870.00(6671.42,11577.62) | 20.45(15.38,26.70) |  | 8011.28(5992.04,10415.86) | 18.57(13.89,24.14) |  | -9.68(-13.03,-6.35) | -9.23(-12.59,-5.88) | -0.28(-0.30,-0.25) |
| Micronesia (Federated States of) | 17.08(12.47,22.92) | 29.70(21.69,39.86) |  | 11.13(8.08,15.29) | 26.86(19.49,36.90) |  | -34.81(-42.76,-24.31) | -9.55(-20.59,5.00) | -0.31(-0.32,-0.29) |
| Monaco | 1.53(1.18,1.97) | 31.55(24.32,40.77) |  | 2.21(1.68,2.87) | 32.65(24.77,42.46) |  | 44.61(25.52,65.77) | 3.49(-10.18,18.63) | 0.05(0.01,0.09) |
| Mongolia | 433.90(311.80,576.45) | 38.23(27.47,50.78) |  | 511.98(382.50,680.93) | 39.00(29.14,51.87) |  | 17.99(1.97,36.66) | 2.03(-11.82,18.17) | 0.22(0.08,0.35) |
| Montenegro | 40.75(30.81,53.64) | 19.00(14.36,25.01) |  | 31.68(24.02,42.05) | 21.07(15.97,27.96) |  | -22.26(-32.47,-9.68) | 10.89(-3.68,28.83) | 0.19(0.13,0.25) |
| Morocco | 3363.51(2469.75,4508.24) | 26.95(19.79,36.12) |  | 3251.44(2437.91,4331.41) | 25.17(18.88,33.54) |  | -3.33(-16.41,11.54) | -6.59(-19.23,7.78) | -0.20(-0.21,-0.19) |
| Mozambique | 2740.66(1952.19,3658.76) | 36.20(25.79,48.33) |  | 6042.62(4321.51,8028.25) | 34.03(24.34,45.22) |  | 120.48(90.43,156.48) | -5.98(-18.80,9.37) | -0.18(-0.23,-0.14) |
| Myanmar | 5195.12(3850.74,6783.30) | 27.18(20.15,35.49) |  | 5253.65(3893.14,6837.92) | 25.30(18.75,32.93) |  | 1.13(-12.48,15.96) | -6.94(-19.46,6.71) | -0.22(-0.24,-0.19) |
| Namibia | 251.88(183.96,345.53) | 33.07(24.15,45.36) |  | 324.06(237.52,429.86) | 30.25(22.17,40.12) |  | 28.66(11.54,48.31) | -8.53(-20.70,5.44) | -0.24(-0.26,-0.23) |
| Nauru | 1.58(1.14,2.12) | 30.26(21.92,40.76) |  | 1.45(1.06,1.96) | 28.35(20.60,38.31) |  | -7.79(-19.94,7.08) | -6.34(-18.68,8.77) | -0.17(-0.23,-0.10) |
| Nepal | 3135.67(2261.14,4178.15) | 30.30(21.85,40.37) |  | 3169.73(2332.47,4224.51) | 25.45(18.73,33.92) |  | 1.09(-15.01,18.00) | -16.00(-29.38,-1.95) | -0.66(-0.71,-0.60) |
| Netherlands | 1707.34(1289.32,2226.81) | 44.54(33.64,58.09) |  | 1728.27(1304.47,2191.30) | 46.82(35.34,59.37) |  | 1.23(-13.26,16.04) | 5.12(-9.92,20.50) | 0.11(0.07,0.16) |
| New Zealand | 347.67(264.16,453.46) | 31.58(23.99,41.18) |  | 438.69(331.39,567.95) | 33.66(25.42,43.57) |  | 26.18(14.65,39.02) | 6.59(-3.15,17.44) | 0.15(0.11,0.18) |
| Nicaragua | 420.95(315.52,558.84) | 18.71(14.02,24.83) |  | 452.30(341.84,596.01) | 17.40(13.15,22.93) |  | 7.45(-6.02,22.69) | -6.98(-18.64,6.22) | -0.22(-0.26,-0.18) |
| Niger | 1616.91(1179.00,2164.90) | 33.33(24.30,44.62) |  | 4923.11(3605.04,6583.45) | 31.59(23.13,42.24) |  | 204.48(158.74,252.53) | -5.23(-19.46,9.73) | -0.24(-0.29,-0.19) |
| Nigeria | 14860.55(10999.96,19369.10) | 30.63(22.67,39.92) |  | 38006.31(28255.61,49796.15) | 29.75(22.11,38.97) |  | 155.75(148.02,163.69) | -2.88(-5.82,0.13) | -0.10(-0.19,-0.01) |
| Niue | 0.25(0.18,0.34) | 24.55(17.97,32.81) |  | 0.12(0.09,0.16) | 23.24(17.21,31.08) |  | -51.10(-57.31,-43.10) | -5.33(-17.36,10.17) | -0.10(-0.17,-0.03) |
| North Macedonia | 136.57(101.34,180.68) | 19.60(14.54,25.92) |  | 91.63(68.46,120.68) | 20.52(15.33,27.02) |  | -32.91(-41.48,-24.35) | 4.71(-8.67,18.06) | 0.01(-0.09,0.10) |
| Northern Mariana Islands | 3.22(2.39,4.30) | 19.87(14.76,26.54) |  | 3.02(2.23,3.96) | 20.15(14.87,26.41) |  | -6.23(-18.10,7.65) | 1.40(-11.44,16.41) | 0.01(-0.07,0.08) |
| Norway | 291.67(222.65,375.66) | 26.22(20.01,33.77) |  | 327.65(254.22,422.91) | 26.39(20.48,34.07) |  | 12.34(7.38,17.41) | 0.67(-3.77,5.22) | -0.02(-0.04,-0.00) |
| Oman | 276.16(201.31,371.35) | 27.55(20.09,37.05) |  | 362.09(269.58,478.59) | 24.27(18.07,32.08) |  | 31.12(10.92,51.94) | -11.91(-25.47,2.08) | -0.29(-0.40,-0.18) |
| Pakistan | 20070.13(14844.72,26235.60) | 32.92(24.35,43.04) |  | 33551.63(25125.06,43822.04) | 30.72(23.00,40.12) |  | 67.17(52.77,85.30) | -6.69(-14.73,3.43) | -0.26(-0.28,-0.24) |
| Palau | 1.54(1.13,2.05) | 24.99(18.35,33.22) |  | 1.07(0.79,1.44) | 24.25(17.92,32.55) |  | -30.31(-39.12,-19.20) | -2.97(-15.24,12.49) | -0.02(-0.05,0.02) |
| Palestine | 293.88(217.76,398.53) | 24.62(18.24,33.39) |  | 521.98(390.99,676.66) | 21.68(16.24,28.10) |  | 77.62(55.52,105.26) | -11.95(-22.91,1.75) | -0.45(-0.47,-0.43) |
| Panama | 189.07(139.24,251.26) | 17.39(12.81,23.11) |  | 248.32(186.98,327.52) | 16.48(12.41,21.73) |  | 31.34(11.85,51.78) | -5.28(-19.33,9.47) | -0.16(-0.18,-0.15) |
| Papua New Guinea | 670.32(495.45,887.09) | 31.49(23.28,41.67) |  | 1557.43(1119.65,2086.57) | 31.79(22.85,42.59) |  | 132.34(103.01,168.39) | 0.95(-11.79,16.62) | 0.06(0.04,0.07) |
| Paraguay | 298.51(222.04,393.40) | 14.43(10.73,19.01) |  | 370.82(277.78,488.98) | 13.88(10.40,18.31) |  | 24.22(8.36,43.01) | -3.76(-16.05,10.80) | -0.13(-0.14,-0.11) |
| Peru | 2535.79(1810.38,3371.42) | 23.81(17.00,31.66) |  | 2683.47(1997.30,3604.84) | 21.45(15.96,28.81) |  | 5.82(-8.03,24.77) | -9.92(-21.71,6.22) | -0.37(-0.41,-0.33) |
| Philippines | 8162.84(6007.35,10575.18) | 25.47(18.74,32.99) |  | 10941.45(8215.32,14268.93) | 24.44(18.35,31.87) |  | 34.04(30.61,37.34) | -4.05(-6.51,-1.69) | -0.09(-0.12,-0.07) |
| Poland | 1854.78(1348.36,2508.16) | 14.90(10.83,20.15) |  | 1211.46(879.31,1611.44) | 15.74(11.42,20.93) |  | -34.68(-37.50,-32.34) | 5.62(1.07,9.41) | 0.25(0.11,0.38) |
| Portugal | 1377.25(1038.89,1805.74) | 46.20(34.85,60.58) |  | 938.29(697.87,1222.57) | 48.95(36.41,63.78) |  | -31.87(-41.39,-22.17) | 5.95(-8.85,21.05) | 0.17(0.15,0.19) |
| Puerto Rico | 232.08(172.02,307.24) | 17.42(12.91,23.06) |  | 104.60(77.71,139.06) | 16.01(11.89,21.28) |  | -54.93(-60.66,-48.39) | -8.13(-19.80,5.21) | -0.30(-0.32,-0.28) |
| Qatar | 37.07(27.87,48.32) | 24.22(18.21,31.57) |  | 129.31(97.37,166.97) | 21.66(16.31,27.96) |  | 248.79(197.20,300.70) | -10.58(-23.81,2.73) | -0.35(-0.38,-0.32) |
| Republic of Korea | 2763.56(2011.30,3585.72) | 17.35(12.63,22.51) |  | 1373.98(1042.49,1805.74) | 16.39(12.44,21.54) |  | -50.28(-56.64,-41.42) | -5.54(-17.62,11.30) | -0.32(-0.54,-0.10) |
| Republic of Moldova | 569.08(418.35,759.18) | 35.99(26.46,48.01) |  | 244.82(181.40,324.17) | 35.16(26.05,46.56) |  | -56.98(-63.27,-50.34) | -2.29(-16.59,12.80) | 0.13(0.01,0.26) |
| Romania | 1629.96(1204.83,2163.92) | 21.72(16.06,28.84) |  | 906.74(678.11,1211.84) | 22.44(16.78,29.99) |  | -44.37(-51.56,-35.06) | 3.29(-10.05,20.58) | 0.11(0.05,0.18) |
| Russian Federation | 17960.98(13458.88,23339.53) | 39.75(29.79,51.65) |  | 13259.33(9979.69,17316.48) | 39.24(29.53,51.25) |  | -26.18(-28.15,-24.17) | -1.28(-3.92,1.40) | 0.23(0.11,0.35) |
| Rwanda | 1454.55(1031.79,1915.68) | 35.15(24.93,46.29) |  | 2015.76(1477.47,2629.72) | 31.31(22.95,40.85) |  | 38.58(18.70,60.85) | -10.91(-23.69,3.40) | -0.39(-0.41,-0.37) |
| Saint Kitts and Nevis | 3.76(2.76,4.96) | 20.46(15.03,27.01) |  | 2.60(1.91,3.39) | 18.50(13.58,24.14) |  | -30.79(-40.16,-19.50) | -9.58(-21.83,5.17) | -0.36(-0.40,-0.32) |
| Saint Lucia | 12.53(9.42,16.65) | 18.77(14.11,24.93) |  | 7.60(5.60,10.04) | 18.10(13.35,23.93) |  | -39.38(-47.32,-30.44) | -3.52(-16.16,10.71) | -0.12(-0.17,-0.07) |
| Saint Vincent and the Grenadines | 10.14(7.44,13.30) | 18.86(13.84,24.74) |  | 6.22(4.66,8.21) | 18.44(13.83,24.35) |  | -38.68(-47.36,-29.51) | -2.21(-16.05,12.41) | -0.05(-0.07,-0.02) |
| Samoa | 22.90(16.71,30.28) | 25.00(18.25,33.05) |  | 24.51(17.73,32.57) | 24.28(17.56,32.27) |  | 7.06(-5.82,22.93) | -2.85(-14.54,11.55) | -0.08(-0.10,-0.07) |
| San Marino | 1.68(1.26,2.17) | 28.10(21.10,36.24) |  | 1.93(1.47,2.53) | 30.87(23.58,40.53) |  | 14.55(0.23,32.02) | 9.84(-3.89,26.60) | 0.39(0.33,0.45) |
| Sao Tome and Principe | 19.13(13.87,25.11) | 27.37(19.85,35.92) |  | 25.39(19.11,33.58) | 24.94(18.78,32.98) |  | 32.73(10.37,53.27) | -8.89(-24.24,5.22) | -0.29(-0.33,-0.25) |
| Saudi Arabia | 2250.57(1677.26,2964.48) | 27.41(20.43,36.10) |  | 2512.03(1850.62,3303.24) | 24.79(18.26,32.60) |  | 11.62(-2.57,28.06) | -9.55(-21.05,3.77) | -0.31(-0.36,-0.27) |
| Senegal | 1348.04(972.45,1806.66) | 30.31(21.87,40.63) |  | 2378.14(1779.51,3185.62) | 29.30(21.93,39.25) |  | 76.41(51.60,103.72) | -3.33(-16.93,11.64) | -0.04(-0.08,-0.01) |
| Serbia | 564.79(416.61,751.33) | 19.47(14.36,25.90) |  | 395.76(295.25,523.48) | 21.12(15.76,27.94) |  | -29.93(-38.38,-21.29) | 8.48(-4.60,21.86) | 0.23(0.17,0.29) |
| Seychelles | 6.91(5.03,9.34) | 22.12(16.10,29.91) |  | 6.64(4.87,9.02) | 21.75(15.95,29.54) |  | -3.91(-15.67,10.87) | -1.67(-13.70,13.47) | -0.05(-0.06,-0.04) |
| Sierra Leone | 740.81(547.66,1023.86) | 33.59(24.83,46.42) |  | 1382.49(1013.29,1838.32) | 30.38(22.27,40.40) |  | 86.62(62.10,114.58) | -9.55(-21.43,4.01) | -0.32(-0.34,-0.30) |
| Singapore | 150.08(114.21,194.86) | 16.30(12.40,21.16) |  | 167.44(126.37,220.11) | 16.05(12.11,21.10) |  | 11.56(-2.23,25.95) | -1.53(-13.70,11.17) | -0.24(-0.47,-0.02) |
| Slovakia | 354.92(265.01,464.25) | 20.22(15.10,26.45) |  | 242.85(177.95,321.13) | 21.66(15.87,28.64) |  | -31.57(-40.47,-19.21) | 7.13(-6.79,26.49) | 0.22(0.15,0.29) |
| Slovenia | 103.04(76.38,134.04) | 18.51(13.72,24.07) |  | 80.48(58.99,104.83) | 19.88(14.57,25.89) |  | -21.90(-32.22,-11.08) | 7.42(-6.77,22.29) | 0.15(0.09,0.21) |
| Solomon Islands | 47.09(35.19,63.02) | 24.20(18.08,32.38) |  | 77.77(56.90,104.48) | 23.51(17.20,31.59) |  | 65.15(45.35,88.99) | -2.82(-14.47,11.20) | -0.03(-0.07,0.00) |
| Somalia | 1917.69(1396.24,2552.52) | 40.52(29.50,53.94) |  | 5006.52(3654.88,6634.00) | 39.16(28.59,51.89) |  | 161.07(127.40,199.42) | -3.36(-15.83,10.84) | -0.05(-0.08,-0.01) |
| South Africa | 5749.58(4259.60,7669.63) | 32.63(24.17,43.52) |  | 6290.36(4670.91,8377.81) | 31.58(23.45,42.06) |  | 9.41(3.92,16.04) | -3.21(-8.06,2.67) | -0.09(-0.11,-0.06) |
| South Sudan | 1126.43(814.31,1496.52) | 34.28(24.78,45.54) |  | 1804.74(1330.16,2412.37) | 32.89(24.24,43.96) |  | 60.22(39.22,86.05) | -4.05(-16.63,11.42) | -0.12(-0.17,-0.07) |
| Spain | 1946.67(1560.16,2427.03) | 17.46(14.00,21.77) |  | 1833.16(1465.53,2290.43) | 20.81(16.64,26.01) |  | -5.83(-18.05,7.29) | 19.18(3.72,35.79) | 1.51(1.25,1.77) |
| Sri Lanka | 1602.20(1177.11,2121.10) | 22.13(16.26,29.30) |  | 1399.73(1030.56,1839.99) | 20.29(14.94,26.67) |  | -12.64(-23.91,4.03) | -8.33(-20.16,9.15) | -0.26(-0.29,-0.23) |
| Sudan | 3411.29(2496.66,4551.68) | 30.88(22.60,41.21) |  | 5786.05(4352.81,7643.55) | 27.07(20.36,35.76) |  | 69.61(45.48,95.00) | -12.35(-24.82,0.77) | -0.39(-0.42,-0.36) |
| Suriname | 34.39(24.79,46.73) | 20.11(14.50,27.33) |  | 37.69(27.37,50.61) | 19.85(14.41,26.66) |  | 9.60(-5.80,26.70) | -1.29(-15.16,14.11) | -0.01(-0.03,0.00) |
| Sweden | 470.37(354.86,607.56) | 22.32(16.84,28.83) |  | 544.77(417.89,707.68) | 22.56(17.30,29.30) |  | 15.82(4.60,27.75) | 1.03(-8.75,11.44) | -0.19(-0.39,0.01) |
| Switzerland | 460.62(347.77,604.53) | 29.21(22.05,38.33) |  | 550.05(416.58,722.71) | 31.21(23.63,41.00) |  | 19.42(6.81,37.42) | 6.85(-4.43,22.96) | 0.23(0.20,0.25) |
| Syrian Arab Republic | 1869.35(1384.13,2489.48) | 25.43(18.83,33.86) |  | 1146.53(867.94,1509.55) | 21.08(15.95,27.75) |  | -38.67(-46.63,-29.20) | -17.11(-27.87,-4.32) | -0.60(-0.65,-0.55) |
| Taiwan (Province of China) | 1454.91(1086.24,1894.84) | 19.91(14.86,25.92) |  | 726.92(559.55,932.28) | 17.97(13.83,23.05) |  | -50.04(-56.59,-43.39) | -9.73(-21.58,2.27) | -0.38(-0.44,-0.31) |
| Tajikistan | 962.06(708.96,1279.96) | 33.47(24.66,44.53) |  | 1542.60(1114.58,2012.65) | 34.63(25.02,45.18) |  | 60.34(39.07,84.71) | 3.45(-10.27,19.18) | 0.21(0.12,0.30) |
| Thailand | 5059.97(3750.39,6680.40) | 22.24(16.49,29.37) |  | 2803.91(2042.20,3805.75) | 20.54(14.96,27.88) |  | -44.59(-51.25,-36.32) | -7.64(-18.75,6.13) | -0.32(-0.35,-0.29) |
| Timor-Leste | 110.17(81.73,147.21) | 27.09(20.10,36.20) |  | 163.28(121.65,215.99) | 23.82(17.75,31.51) |  | 48.21(29.90,68.81) | -12.07(-22.93,0.15) | -0.51(-0.55,-0.47) |
| Togo | 701.76(509.63,918.87) | 32.60(23.67,42.68) |  | 1295.80(940.88,1732.57) | 31.03(22.53,41.49) |  | 84.65(59.25,112.02) | -4.80(-17.89,9.31) | -0.12(-0.17,-0.08) |
| Tokelau | 0.19(0.14,0.25) | 24.73(18.04,32.80) |  | 0.12(0.08,0.16) | 22.82(16.44,30.85) |  | -37.75(-45.94,-28.09) | -7.72(-19.85,6.60) | -0.22(-0.29,-0.15) |
| Tonga | 12.27(9.05,16.26) | 22.99(16.96,30.47) |  | 11.34(8.36,15.01) | 22.88(16.86,30.28) |  | -7.55(-18.62,6.35) | -0.51(-12.41,14.45) | 0.01(-0.01,0.03) |
| Trinidad and Tobago | 101.99(75.16,137.03) | 19.63(14.47,26.38) |  | 68.26(50.73,89.77) | 18.75(13.94,24.66) |  | -33.07(-41.70,-23.77) | -4.48(-16.79,8.79) | -0.04(-0.11,0.02) |
| Tunisia | 913.65(688.26,1196.72) | 22.90(17.25,29.99) |  | 794.48(589.47,1067.11) | 22.16(16.44,29.76) |  | -13.04(-23.85,0.56) | -3.23(-15.26,11.90) | -0.04(-0.10,0.02) |
| Turkey | 6076.94(4539.25,7978.76) | 22.78(17.01,29.90) |  | 5010.79(3760.33,6512.95) | 20.28(15.22,26.36) |  | -17.54(-28.60,-5.51) | -10.97(-22.91,2.02) | -0.40(-0.42,-0.38) |
| Turkmenistan | 629.94(466.91,837.19) | 33.41(24.77,44.41) |  | 655.83(473.88,876.16) | 33.45(24.17,44.69) |  | 4.11(-8.27,20.08) | 0.11(-11.80,15.47) | 0.04(-0.01,0.09) |
| Tuvalu | 1.31(0.97,1.78) | 30.75(22.76,41.58) |  | 1.31(0.95,1.76) | 26.67(19.32,35.82) |  | -0.27(-14.79,15.65) | -13.26(-25.89,0.59) | -0.44(-0.48,-0.41) |
| Uganda | 3610.98(2634.68,4833.37) | 35.07(25.58,46.94) |  | 8243.57(6099.48,11212.45) | 33.05(24.45,44.95) |  | 128.29(95.88,165.63) | -5.75(-19.13,9.67) | -0.20(-0.22,-0.18) |
| Ukraine | 5739.30(4276.06,7518.79) | 38.13(28.41,49.95) |  | 3340.14(2434.16,4454.28) | 39.55(28.82,52.74) |  | -41.80(-49.53,-33.16) | 3.72(-10.04,19.13) | 0.31(0.21,0.41) |
| United Arab Emirates | 194.13(142.05,262.23) | 27.46(20.09,37.09) |  | 443.18(328.16,588.81) | 26.14(19.36,34.74) |  | 128.29(95.91,163.88) | -4.78(-18.29,10.06) | -0.15(-0.20,-0.09) |
| United Kingdom | 4267.12(3267.23,5476.15) | 28.77(22.03,36.92) |  | 4650.39(3578.01,5919.86) | 29.71(22.86,37.82) |  | 8.98(6.46,11.38) | 3.27(0.88,5.54) | 0.18(0.14,0.22) |
| United Republic of Tanzania | 4773.79(3421.28,6316.81) | 31.99(22.93,42.33) |  | 9328.09(6861.51,12586.88) | 30.23(22.24,40.79) |  | 95.40(68.46,129.55) | -5.50(-18.53,11.01) | -0.19(-0.22,-0.17) |
| United States of America | 21179.19(15202.28,28651.24) | 28.60(20.53,38.69) |  | 22213.65(15853.47,30179.59) | 27.32(19.50,37.12) |  | 4.88(2.21,8.38) | -4.49(-6.92,-1.31) | -0.34(-0.44,-0.24) |
| United States Virgin Islands | 8.10(5.92,10.99) | 19.42(14.19,26.34) |  | 3.45(2.57,4.62) | 18.96(14.13,25.34) |  | -57.37(-63.32,-50.05) | -2.40(-16.02,14.36) | -0.07(-0.09,-0.04) |
| Uruguay | 125.21(91.47,163.65) | 11.63(8.49,15.19) |  | 102.67(75.14,137.59) | 11.37(8.32,15.23) |  | -18.00(-28.87,-6.13) | -2.23(-15.20,11.92) | -0.26(-0.48,-0.04) |
| Uzbekistan | 3470.41(2543.04,4664.16) | 32.51(23.82,43.69) |  | 4259.42(3141.53,5602.33) | 33.78(24.91,44.43) |  | 22.74(9.20,39.23) | 3.91(-7.55,17.87) | 0.17(0.07,0.28) |
| Vanuatu | 24.85(17.78,33.56) | 29.78(21.30,40.22) |  | 42.05(30.83,57.07) | 28.52(20.91,38.70) |  | 69.21(47.86,93.46) | -4.23(-16.31,9.50) | -0.10(-0.12,-0.09) |
| Venezuela (Bolivarian Republic of) | 1883.06(1390.92,2481.15) | 20.75(15.33,27.34) |  | 1676.04(1233.28,2239.38) | 19.15(14.09,25.59) |  | -10.99(-23.43,2.55) | -7.70(-20.59,6.35) | -0.22(-0.26,-0.19) |
| Viet Nam | 7523.56(5587.37,9959.50) | 22.35(16.60,29.58) |  | 6580.42(4923.43,8701.80) | 20.81(15.57,27.52) |  | -12.54(-23.66,0.09) | -6.87(-18.72,6.57) | -0.17(-0.25,-0.10) |
| Yemen | 2713.72(1984.48,3559.32) | 32.28(23.60,42.33) |  | 4988.90(3655.99,6503.40) | 28.55(20.92,37.22) |  | 83.84(59.05,114.74) | -11.53(-23.46,3.34) | -0.38(-0.39,-0.36) |
| Zambia | 1654.32(1216.48,2215.33) | 35.19(25.88,47.13) |  | 3406.33(2523.00,4502.99) | 32.58(24.13,43.07) |  | 105.91(78.91,139.38) | -7.42(-19.56,7.63) | -0.25(-0.30,-0.20) |
| Zimbabwe | 2029.29(1467.90,2773.99) | 33.70(24.37,46.06) |  | 2744.38(2016.14,3591.60) | 34.37(25.25,44.98) |  | 35.24(16.10,56.60) | 2.01(-12.43,18.12) | 0.24(0.17,0.31) |
