# Supplementary figures and images for: The hidden in plain sight: global, regional, and national trends in the pediatric burden of Klinefelter syndrome, 1990–2021
Source: Front Genet. 2025 Sep 16;16:1639699. doi: 10.3389/fgene.2025.1639699 (PMC12479305; doi:10.3389/fgene.2025.1639699)

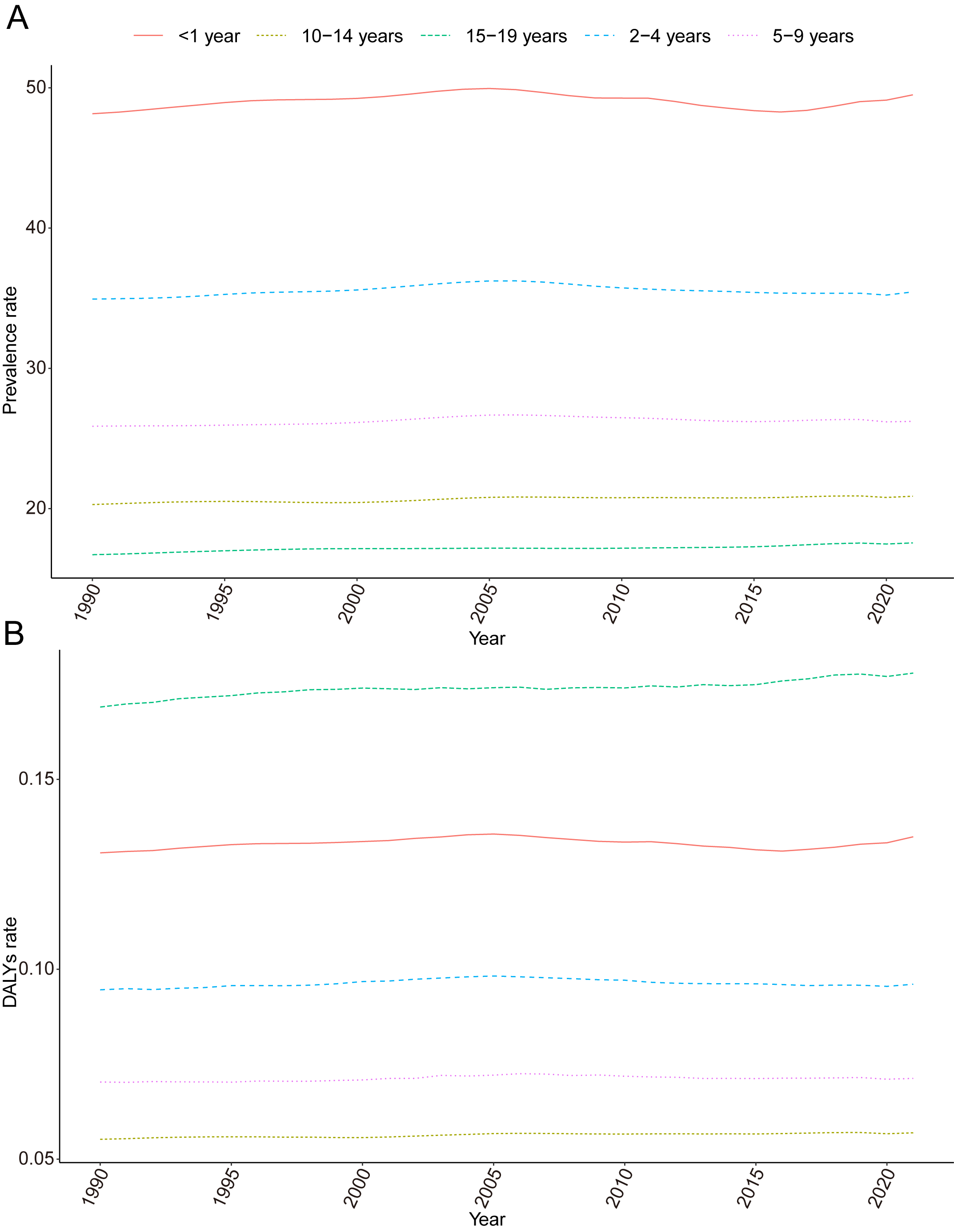

Supplement: Supplementary file 2 [file Image1.tif]
