# Supplementary material for: The hidden in plain sight: global, regional, and national trends in the pediatric burden of Klinefelter syndrome, 1990–2021
Source: Front Genet. 2025 Sep 16;16:1639699. doi: 10.3389/fgene.2025.1639699 (PMC12479305; doi:10.3389/fgene.2025.1639699)
Supplement: Supplementary file 3 [file Table2.docx]

Table S2. DALYs of Klinefelter syndrome in children between 1990 and 2021 at the national levels

| location | 1990 | |  | 2021 | |  | 1990-2021 | | |
| --- | --- | --- | --- | --- | --- | --- | --- | --- | --- |
|  | DALYs cases | DALY rate |  | DALYs cases | DALY rate |  | Cases change | Rate change | EAPC |
| Afghanistan | 7.01(3.13,14.09) | 0.13(0.06,0.25) |  | 21.26(8.95,42.83) | 0.12(0.05,0.24) |  | 203.02(102.24,366.98) | -5.00(-36.60,46.40) | -0.26(-0.33,-0.20) |
| Albania | 1.22(0.54,2.52) | 0.08(0.04,0.17) |  | 0.55(0.24,1.14) | 0.09(0.04,0.19) |  | -54.44(-68.08,-32.51) | 6.76(-25.22,58.13) | 0.33(0.23,0.44) |
| Algeria | 13.19(6.31,26.55) | 0.10(0.05,0.20) |  | 14.92(6.88,30.59) | 0.09(0.04,0.19) |  | 13.17(-19.23,61.54) | -7.01(-33.63,32.74) | -0.22(-0.33,-0.10) |
| American Samoa | 0.02(0.01,0.05) | 0.09(0.04,0.20) |  | 0.02(0.01,0.04) | 0.10(0.04,0.21) |  | -19.66(-48.05,19.97) | 0.48(-35.02,50.06) | 0.01(-0.02,0.05) |
| Andorra | 0.02(0.01,0.04) | 0.13(0.06,0.27) |  | 0.02(0.01,0.04) | 0.14(0.06,0.30) |  | 14.80(-26.96,80.24) | 7.46(-31.63,68.71) | 0.27(0.16,0.39) |
| Angola | 7.08(3.05,14.91) | 0.12(0.05,0.26) |  | 20.97(9.90,40.71) | 0.11(0.05,0.22) |  | 196.16(92.21,340.32) | -8.56(-40.65,35.95) | -0.28(-0.31,-0.24) |
| Antigua and Barbuda | 0.02(0.01,0.03) | 0.07(0.03,0.14) |  | 0.02(0.01,0.03) | 0.07(0.03,0.15) |  | -0.25(-32.79,51.26) | 2.00(-31.28,54.66) | 0.20(0.12,0.28) |
| Argentina | 4.32(2.07,8.56) | 0.03(0.02,0.07) |  | 4.64(2.27,9.23) | 0.03(0.02,0.07) |  | 7.40(-7.23,24.87) | 2.03(-11.86,18.64) | -0.16(-0.43,0.11) |
| Armenia | 1.45(0.68,2.86) | 0.11(0.05,0.22) |  | 0.87(0.37,1.74) | 0.11(0.05,0.23) |  | -40.37(-62.44,-7.75) | 3.76(-34.65,60.51) | 0.24(0.11,0.38) |
| Australia | 3.26(1.42,6.70) | 0.06(0.03,0.13) |  | 3.73(1.64,7.54) | 0.06(0.03,0.12) |  | 14.37(-25.60,73.72) | -5.18(-38.32,44.03) | -0.27(-0.45,-0.09) |
| Austria | 1.99(0.89,4.25) | 0.11(0.05,0.23) |  | 2.01(0.89,3.95) | 0.11(0.05,0.22) |  | 0.74(-29.45,47.98) | 7.53(-24.69,57.96) | 0.40(0.33,0.48) |
| Azerbaijan | 3.69(1.63,7.04) | 0.12(0.05,0.22) |  | 3.63(1.60,7.26) | 0.12(0.05,0.24) |  | -1.56(-37.57,52.54) | 1.13(-35.86,56.71) | 0.29(0.16,0.42) |
| Bahamas | 0.09(0.04,0.18) | 0.08(0.04,0.17) |  | 0.09(0.04,0.19) | 0.08(0.04,0.17) |  | 9.65(-23.29,69.14) | 2.42(-28.35,57.98) | 0.05(-0.01,0.12) |
| Bahrain | 0.18(0.08,0.37) | 0.09(0.04,0.19) |  | 0.39(0.18,0.80) | 0.10(0.04,0.20) |  | 118.88(48.82,216.47) | 8.54(-26.20,56.93) | 0.21(0.10,0.32) |
| Bangladesh | 61.22(25.50,129.70) | 0.10(0.04,0.21) |  | 57.89(27.71,117.44) | 0.09(0.05,0.19) |  | -5.43(-37.79,45.31) | -6.41(-38.43,43.81) | -0.25(-0.30,-0.20) |
| Barbados | 0.06(0.03,0.12) | 0.07(0.03,0.14) |  | 0.05(0.02,0.10) | 0.07(0.03,0.15) |  | -19.49(-50.06,26.14) | 2.98(-36.13,61.34) | 0.05(-0.00,0.10) |
| Belarus | 4.43(1.98,9.24) | 0.14(0.06,0.29) |  | 2.81(1.15,5.79) | 0.14(0.06,0.29) |  | -36.51(-63.13,0.93) | -1.63(-42.88,56.38) | 0.05(-0.15,0.24) |
| Belgium | 4.81(2.13,9.68) | 0.19(0.09,0.39) |  | 5.03(2.20,10.43) | 0.20(0.09,0.41) |  | 4.47(-34.03,65.93) | 1.49(-35.92,61.20) | 0.09(0.05,0.13) |
| Belize | 0.08(0.03,0.15) | 0.07(0.03,0.15) |  | 0.13(0.05,0.27) | 0.08(0.03,0.16) |  | 71.87(16.40,154.54) | 4.29(-29.37,54.45) | 0.06(0.03,0.09) |
| Benin | 3.17(1.48,6.44) | 0.11(0.05,0.22) |  | 8.11(3.62,16.93) | 0.11(0.05,0.23) |  | 156.01(69.95,301.36) | -2.25(-35.11,53.24) | -0.13(-0.16,-0.09) |
| Bermuda | 0.01(0.00,0.02) | 0.07(0.03,0.14) |  | 0.01(0.00,0.02) | 0.07(0.03,0.13) |  | -28.54(-55.07,7.88) | -1.16(-37.87,49.21) | 0.02(-0.06,0.10) |
| Bhutan | 0.36(0.16,0.71) | 0.11(0.05,0.21) |  | 0.25(0.11,0.50) | 0.10(0.04,0.20) |  | -30.14(-54.22,1.72) | -6.67(-38.85,35.88) | -0.24(-0.27,-0.20) |
| Bolivia (Plurinational State of) | 3.29(1.55,6.49) | 0.10(0.05,0.19) |  | 4.30(1.96,8.87) | 0.09(0.04,0.20) |  | 30.60(-7.53,97.68) | -3.81(-31.89,45.60) | -0.12(-0.16,-0.08) |
| Bosnia and Herzegovina | 1.29(0.57,2.66) | 0.09(0.04,0.18) |  | 0.60(0.26,1.24) | 0.09(0.04,0.19) |  | -53.61(-68.37,-31.75) | 3.36(-29.54,52.05) | 0.17(0.10,0.24) |
| Botswana | 0.93(0.41,1.89) | 0.13(0.06,0.26) |  | 1.18(0.50,2.38) | 0.13(0.05,0.26) |  | 27.36(-21.93,104.42) | 2.85(-36.96,65.06) | 0.07(0.02,0.12) |
| Brazil | 40.84(20.01,78.86) | 0.06(0.03,0.12) |  | 40.99(20.04,81.30) | 0.06(0.03,0.13) |  | 0.37(-10.66,15.38) | 5.51(-6.08,21.29) | 0.19(0.16,0.23) |
| Brunei Darussalam | 0.09(0.04,0.18) | 0.08(0.03,0.15) |  | 0.11(0.05,0.22) | 0.08(0.04,0.17) |  | 22.78(-14.32,79.38) | 9.36(-23.68,59.77) | 0.31(0.06,0.55) |
| Bulgaria | 2.14(0.89,4.61) | 0.09(0.04,0.20) |  | 1.26(0.57,2.57) | 0.10(0.04,0.20) |  | -41.06(-59.76,-14.90) | 7.62(-26.53,55.38) | 0.05(-0.08,0.19) |
| Burkina Faso | 6.59(2.91,13.13) | 0.12(0.05,0.23) |  | 14.40(6.38,29.76) | 0.11(0.05,0.23) |  | 118.44(35.45,244.47) | -3.50(-40.16,52.18) | -0.13(-0.17,-0.08) |
| Burundi | 3.93(1.73,7.83) | 0.12(0.05,0.25) |  | 8.76(3.97,18.35) | 0.12(0.05,0.25) |  | 122.70(49.01,248.77) | -3.41(-35.37,51.27) | -0.09(-0.12,-0.06) |
| Cabo Verde | 0.19(0.08,0.36) | 0.10(0.04,0.18) |  | 0.19(0.08,0.37) | 0.10(0.04,0.19) |  | 1.52(-30.95,54.13) | 2.43(-30.33,55.51) | 0.14(0.06,0.21) |
| Cambodia | 5.60(2.43,11.67) | 0.10(0.04,0.20) |  | 6.25(2.89,13.68) | 0.09(0.04,0.21) |  | 11.60(-25.21,74.64) | -4.19(-35.79,49.94) | -0.08(-0.13,-0.03) |
| Cameroon | 6.77(2.91,13.93) | 0.11(0.05,0.23) |  | 18.85(8.17,37.43) | 0.11(0.05,0.22) |  | 178.26(85.33,351.40) | -2.26(-34.90,58.55) | -0.08(-0.12,-0.05) |
| Canada | 10.28(4.45,21.37) | 0.13(0.06,0.28) |  | 11.05(5.03,23.40) | 0.13(0.06,0.28) |  | 7.58(-32.18,68.31) | 0.21(-36.83,56.77) | 0.09(0.04,0.14) |
| Central African Republic | 2.03(0.90,4.21) | 0.14(0.06,0.28) |  | 4.01(1.67,7.94) | 0.14(0.06,0.27) |  | 97.59(24.82,196.26) | 2.22(-35.43,53.26) | 0.00(-0.03,0.04) |
| Chad | 4.05(1.70,8.06) | 0.11(0.05,0.23) |  | 12.41(5.22,26.02) | 0.11(0.05,0.24) |  | 206.23(97.51,381.33) | -1.41(-36.41,54.97) | -0.11(-0.15,-0.07) |
| Chile | 3.55(1.57,7.13) | 0.07(0.03,0.14) |  | 3.24(1.45,6.39) | 0.07(0.03,0.13) |  | -8.67(-40.52,38.08) | -1.56(-35.89,48.82) | -0.07(-0.22,0.09) |
| China | 326.78(157.48,638.56) | 0.07(0.04,0.14) |  | 250.41(124.51,503.95) | 0.07(0.04,0.15) |  | -23.37(-30.44,-16.41) | 2.01(-7.39,11.27) | 0.21(0.04,0.38) |
| Colombia | 11.33(5.24,22.38) | 0.08(0.03,0.15) |  | 10.82(4.91,23.36) | 0.07(0.03,0.16) |  | -4.53(-34.92,37.84) | -1.29(-32.72,42.51) | -0.07(-0.10,-0.03) |
| Comoros | 0.29(0.12,0.59) | 0.11(0.05,0.22) |  | 0.34(0.14,0.70) | 0.11(0.04,0.22) |  | 17.25(-25.02,82.74) | -1.29(-36.88,53.85) | -0.04(-0.09,0.01) |
| Congo | 1.61(0.68,3.48) | 0.12(0.05,0.26) |  | 2.94(1.28,5.84) | 0.12(0.05,0.23) |  | 82.05(17.02,185.95) | -2.88(-37.57,52.55) | -0.15(-0.19,-0.12) |
| Cook Islands | 0.01(0.00,0.02) | 0.09(0.04,0.18) |  | 0.00(0.00,0.01) | 0.08(0.04,0.18) |  | -43.82(-62.29,-17.12) | -6.67(-37.36,37.69) | -0.15(-0.21,-0.09) |
| Costa Rica | 0.92(0.42,1.97) | 0.06(0.03,0.14) |  | 0.90(0.40,1.88) | 0.07(0.03,0.14) |  | -2.66(-34.43,46.05) | 0.84(-32.07,51.30) | 0.05(-0.00,0.10) |
| Croatia | 8.09(3.72,16.16) | 0.12(0.05,0.23) |  | 16.87(7.12,35.44) | 0.12(0.05,0.25) |  | -38.33(-59.87,-7.77) | 1.53(-33.93,51.83) | -0.08(-0.12,-0.04) |
| Cuba | 0.92(0.39,1.93) | 0.07(0.03,0.15) |  | 0.57(0.26,1.11) | 0.07(0.03,0.14) |  | -40.14(-61.19,-8.55) | -8.97(-40.97,39.10) | -0.39(-0.64,-0.14) |
| Cyprus | 2.61(1.19,5.56) | 0.07(0.03,0.15) |  | 1.56(0.71,3.17) | 0.07(0.03,0.13) |  | 12.55(-32.01,82.42) | 2.28(-38.22,65.77) | -0.00(-0.12,0.11) |
| Czechia | 0.35(0.15,0.77) | 0.13(0.06,0.30) |  | 0.39(0.15,0.88) | 0.14(0.05,0.31) |  | -25.41(-50.99,12.89) | 2.24(-32.82,54.75) | 0.13(0.02,0.23) |
| C么te d'Ivoire | 3.32(1.50,6.54) | 0.11(0.05,0.21) |  | 2.47(1.08,5.19) | 0.11(0.05,0.23) |  | 108.43(34.76,211.40) | 0.25(-35.18,49.77) | 0.02(-0.20,0.23) |
| Democratic People's Republic of Korea | 6.50(2.88,13.37) | 0.08(0.04,0.17) |  | 5.88(2.74,12.37) | 0.09(0.04,0.19) |  | -9.63(-38.29,37.77) | 6.62(-27.20,62.54) | 0.30(0.24,0.35) |
| Democratic Republic of the Congo | 26.03(11.31,51.98) | 0.12(0.05,0.24) |  | 55.72(23.88,111.38) | 0.12(0.05,0.23) |  | 114.09(45.47,220.29) | -3.25(-34.26,44.75) | -0.07(-0.12,-0.03) |
| Denmark | 1.74(0.79,3.71) | 0.14(0.06,0.30) |  | 1.83(0.78,4.01) | 0.14(0.06,0.31) |  | 5.08(-38.07,65.94) | 1.51(-40.18,60.30) | 0.08(-0.02,0.19) |
| Djibouti | 0.26(0.11,0.49) | 0.12(0.05,0.22) |  | 0.64(0.28,1.37) | 0.12(0.05,0.26) |  | 143.41(57.85,262.75) | 3.32(-33.00,53.97) | 0.07(0.03,0.11) |
| Dominica | 0.03(0.01,0.05) | 0.08(0.04,0.16) |  | 0.02(0.01,0.03) | 0.08(0.04,0.17) |  | -39.34(-60.25,-9.91) | 2.35(-32.94,51.99) | 0.11(0.04,0.18) |
| Dominican Republic | 2.62(1.15,5.12) | 0.07(0.03,0.15) |  | 2.92(1.31,5.87) | 0.08(0.03,0.15) |  | 11.70(-21.71,61.51) | 0.54(-29.52,45.38) | 0.11(0.07,0.16) |
| Ecuador | 4.86(2.28,9.53) | 0.10(0.05,0.19) |  | 5.95(2.73,12.45) | 0.09(0.04,0.19) |  | 22.36(-17.36,77.74) | -8.32(-38.08,33.18) | -0.27(-0.32,-0.22) |
| Egypt | 28.95(12.66,55.72) | 0.10(0.05,0.20) |  | 44.10(20.46,88.64) | 0.09(0.04,0.19) |  | 52.33(4.09,117.13) | -8.62(-37.56,30.24) | -0.27(-0.33,-0.20) |
| El Salvador | 2.31(1.08,4.82) | 0.08(0.04,0.17) |  | 1.92(0.87,3.86) | 0.08(0.04,0.16) |  | -16.87(-45.64,19.06) | -3.84(-37.12,37.72) | -0.05(-0.13,0.03) |
| Equatorial Guinea | 0.30(0.13,0.62) | 0.12(0.05,0.26) |  | 0.93(0.41,1.91) | 0.12(0.05,0.25) |  | 213.07(99.60,388.55) | -2.44(-37.80,52.24) | -0.01(-0.04,0.03) |
| Eritrea | 2.58(1.06,5.09) | 0.13(0.05,0.26) |  | 4.13(1.86,8.21) | 0.13(0.06,0.26) |  | 59.74(6.39,141.22) | -2.33(-34.95,47.49) | -0.08(-0.11,-0.06) |
| Estonia | 0.64(0.30,1.28) | 0.14(0.07,0.28) |  | 0.39(0.17,0.80) | 0.14(0.06,0.28) |  | -39.15(-62.69,-3.58) | -0.29(-38.85,58.00) | 0.00(-0.16,0.16) |
| Eswatini | 0.61(0.27,1.28) | 0.13(0.06,0.27) |  | 0.71(0.32,1.36) | 0.13(0.06,0.25) |  | 15.57(-24.04,78.98) | 3.06(-32.26,59.61) | 0.13(0.09,0.16) |
| Ethiopia | 36.95(17.44,69.15) | 0.13(0.06,0.23) |  | 68.00(33.27,126.09) | 0.12(0.06,0.22) |  | 84.05(49.37,130.66) | -5.18(-23.05,18.83) | -0.23(-0.25,-0.21) |
| Fiji | 0.32(0.14,0.66) | 0.09(0.04,0.19) |  | 0.32(0.14,0.69) | 0.09(0.04,0.20) |  | -0.71(-33.94,55.35) | 1.58(-32.42,58.93) | 0.01(-0.05,0.07) |
| Finland | 2.11(0.76,4.38) | 0.17(0.06,0.34) |  | 1.97(0.76,4.18) | 0.17(0.07,0.36) |  | -6.70(-45.84,66.74) | 3.07(-40.17,84.19) | -0.01(-0.10,0.09) |
| France | 18.33(8.62,36.79) | 0.11(0.05,0.23) |  | 22.06(9.52,45.30) | 0.14(0.06,0.29) |  | 20.36(-20.52,77.50) | 23.34(-18.55,81.89) | 0.65(0.34,0.95) |
| Gabon | 0.57(0.25,1.17) | 0.11(0.05,0.23) |  | 0.89(0.38,1.81) | 0.11(0.05,0.22) |  | 54.50(-6.75,133.71) | -5.07(-42.70,43.60) | -0.18(-0.22,-0.15) |
| Gambia | 0.61(0.27,1.29) | 0.11(0.05,0.23) |  | 1.39(0.61,2.89) | 0.11(0.05,0.23) |  | 127.33(47.67,272.18) | 0.83(-34.50,65.08) | -0.05(-0.08,-0.02) |
| Georgia | 2.05(0.86,4.27) | 0.11(0.05,0.24) |  | 1.12(0.49,2.28) | 0.12(0.05,0.25) |  | -45.47(-64.71,-18.31) | 5.43(-31.76,57.95) | 0.38(0.26,0.50) |
| Germany | 23.12(10.10,48.43) | 0.13(0.06,0.28) |  | 22.61(9.40,47.79) | 0.14(0.06,0.30) |  | -2.22(-39.93,66.26) | 6.26(-34.72,80.69) | 0.31(0.25,0.36) |
| Ghana | 9.24(4.10,18.23) | 0.11(0.05,0.22) |  | 17.78(7.98,37.08) | 0.11(0.05,0.23) |  | 92.34(20.97,194.61) | -2.62(-38.75,49.16) | -0.06(-0.10,-0.03) |
| Greece | 3.75(1.81,7.98) | 0.13(0.06,0.28) |  | 2.77(1.05,5.94) | 0.15(0.06,0.31) |  | -26.36(-57.51,22.16) | 8.63(-37.32,80.20) | 0.14(0.07,0.21) |
| Greenland | 0.03(0.01,0.07) | 0.18(0.08,0.37) |  | 0.03(0.01,0.05) | 0.17(0.07,0.34) |  | -16.83(-48.85,36.31) | -3.19(-40.46,58.67) | 0.07(-0.00,0.14) |
| Grenada | 0.03(0.01,0.06) | 0.07(0.03,0.14) |  | 0.02(0.01,0.05) | 0.08(0.03,0.16) |  | -22.40(-47.21,13.23) | 8.67(-26.07,58.57) | 0.34(0.27,0.40) |
| Guam | 0.05(0.02,0.10) | 0.09(0.04,0.18) |  | 0.04(0.02,0.09) | 0.09(0.04,0.18) |  | -9.32(-36.19,31.46) | 1.27(-28.74,46.80) | 0.23(0.13,0.32) |
| Guatemala | 4.17(1.93,8.25) | 0.08(0.04,0.17) |  | 5.54(2.36,10.86) | 0.08(0.04,0.16) |  | 32.89(-8.86,103.20) | -1.34(-32.34,50.85) | -0.09(-0.16,-0.02) |
| Guinea | 3.77(1.59,7.77) | 0.11(0.05,0.24) |  | 8.29(3.71,17.30) | 0.11(0.05,0.23) |  | 119.57(44.80,234.07) | -3.30(-36.23,47.12) | -0.10(-0.16,-0.04) |
| Guinea-Bissau | 0.75(0.34,1.57) | 0.13(0.06,0.27) |  | 1.40(0.66,2.84) | 0.12(0.06,0.25) |  | 87.04(18.68,193.39) | -1.66(-37.60,54.26) | -0.05(-0.08,-0.01) |
| Guyana | 0.33(0.15,0.67) | 0.09(0.04,0.17) |  | 0.24(0.11,0.48) | 0.09(0.04,0.17) |  | -27.33(-50.41,7.77) | -1.21(-32.59,46.50) | 0.09(0.00,0.17) |
| Haiti | 2.95(1.35,5.92) | 0.09(0.04,0.18) |  | 5.08(2.32,10.34) | 0.09(0.04,0.18) |  | 71.96(15.28,149.26) | 2.62(-31.21,48.75) | 0.07(0.04,0.10) |
| Honduras | 2.09(0.93,4.03) | 0.08(0.03,0.15) |  | 3.36(1.61,6.62) | 0.08(0.04,0.15) |  | 61.18(6.55,142.22) | 0.54(-33.54,51.08) | -0.01(-0.05,0.02) |
| Hungary | 3.52(1.52,7.34) | 0.12(0.05,0.25) |  | 2.50(1.10,5.41) | 0.13(0.06,0.29) |  | -28.82(-55.12,11.59) | 10.15(-30.55,72.68) | 0.49(0.33,0.65) |
| Iceland | 0.11(0.05,0.23) | 0.13(0.05,0.27) |  | 0.11(0.05,0.23) | 0.13(0.06,0.26) |  | 7.95(-33.33,80.78) | 2.38(-36.76,71.46) | 0.13(0.07,0.18) |
| India | 463.80(231.04,912.54) | 0.11(0.06,0.22) |  | 552.81(274.88,1073.90) | 0.11(0.05,0.21) |  | 19.19(6.86,33.09) | -2.28(-12.39,9.12) | -0.10(-0.13,-0.06) |
| Indonesia | 80.76(39.19,163.04) | 0.09(0.04,0.19) |  | 84.79(41.41,166.68) | 0.09(0.05,0.18) |  | 4.99(-5.54,17.44) | 1.79(-8.42,13.85) | 0.06(0.04,0.08) |
| Iran (Islamic Republic of) | 30.75(15.10,57.62) | 0.10(0.05,0.18) |  | 24.06(11.76,47.97) | 0.09(0.05,0.19) |  | -21.76(-28.65,-14.24) | -4.64(-13.04,4.51) | -0.11(-0.23,0.00) |
| Iraq | 10.70(4.77,21.39) | 0.10(0.05,0.21) |  | 17.11(7.46,37.24) | 0.10(0.04,0.21) |  | 59.85(5.32,136.76) | -6.66(-38.50,38.25) | -0.30(-0.34,-0.26) |
| Ireland | 2.47(1.03,5.11) | 0.19(0.08,0.38) |  | 2.53(1.11,5.42) | 0.19(0.08,0.41) |  | 2.54(-39.33,74.90) | 3.10(-39.00,75.85) | -0.13(-0.24,-0.03) |
| Israel | 2.60(1.05,5.31) | 0.13(0.05,0.26) |  | 4.39(1.89,9.26) | 0.13(0.06,0.27) |  | 69.22(5.65,175.79) | 0.64(-37.16,64.02) | -0.06(-0.09,-0.02) |
| Italy | 19.34(9.35,39.61) | 0.14(0.07,0.29) |  | 14.92(7.10,29.22) | 0.14(0.07,0.28) |  | -22.83(-33.81,-10.85) | 0.29(-13.98,15.86) | -0.14(-0.21,-0.08) |
| Jamaica | 0.72(0.34,1.48) | 0.07(0.03,0.14) |  | 0.59(0.25,1.29) | 0.07(0.03,0.16) |  | -18.64(-49.18,27.05) | 9.01(-31.91,70.23) | 0.31(0.25,0.36) |
| Japan | 28.84(13.74,58.41) | 0.09(0.04,0.18) |  | 18.63(9.26,36.59) | 0.09(0.04,0.17) |  | -35.42(-44.77,-25.70) | 1.48(-13.22,16.74) | 0.04(-0.11,0.18) |
| Jordan | 1.93(0.83,4.16) | 0.09(0.04,0.20) |  | 4.29(1.92,8.69) | 0.09(0.04,0.18) |  | 121.75(49.89,224.67) | -5.60(-36.19,38.21) | -0.20(-0.25,-0.15) |
| Kazakhstan | 8.43(3.91,17.63) | 0.13(0.06,0.27) |  | 8.59(3.60,17.66) | 0.13(0.05,0.26) |  | 1.85(-36.57,61.79) | 0.62(-37.33,59.84) | 0.07(-0.10,0.24) |
| Kenya | 15.49(7.75,30.21) | 0.11(0.06,0.22) |  | 28.47(14.36,55.62) | 0.12(0.06,0.23) |  | 83.77(69.29,102.31) | 3.03(-5.09,13.42) | 0.07(0.05,0.09) |
| Kiribati | 0.04(0.02,0.09) | 0.12(0.05,0.23) |  | 0.06(0.03,0.12) | 0.11(0.05,0.22) |  | 40.66(-8.68,115.40) | -3.54(-37.37,47.73) | -0.06(-0.11,0.00) |
| Kuwait | 0.52(0.25,1.11) | 0.08(0.04,0.16) |  | 0.87(0.41,1.74) | 0.08(0.04,0.16) |  | 67.13(19.53,131.18) | 4.65(-25.15,44.76) | -0.00(-0.06,0.06) |
| Kyrgyzstan | 2.61(1.16,5.69) | 0.12(0.05,0.27) |  | 3.47(1.57,7.10) | 0.12(0.06,0.25) |  | 33.07(-10.43,106.79) | -0.47(-33.00,54.68) | 0.14(0.01,0.27) |
| Lao People's Democratic Republic | 2.27(0.99,4.56) | 0.10(0.04,0.20) |  | 2.92(1.37,5.77) | 0.10(0.05,0.19) |  | 28.51(-14.76,100.03) | -2.55(-35.36,51.68) | -0.09(-0.12,-0.05) |
| Latvia | 1.09(0.47,2.18) | 0.14(0.06,0.29) |  | 0.57(0.22,1.19) | 0.15(0.06,0.31) |  | -47.73(-70.51,-16.03) | 1.60(-42.68,63.22) | 0.18(-0.02,0.39) |
| Lebanon | 1.29(0.55,2.51) | 0.10(0.04,0.19) |  | 1.48(0.70,3.11) | 0.09(0.04,0.19) |  | 14.84(-23.22,65.75) | -7.71(-38.29,33.21) | -0.20(-0.27,-0.14) |
| Lesotho | 1.11(0.47,2.30) | 0.13(0.06,0.28) |  | 1.16(0.49,2.41) | 0.14(0.06,0.29) |  | 4.52(-33.89,64.82) | 4.33(-34.01,64.53) | 0.13(0.08,0.17) |
| Liberia | 1.50(0.68,2.86) | 0.11(0.05,0.21) |  | 3.00(1.32,6.15) | 0.11(0.05,0.22) |  | 99.31(22.43,215.59) | -2.41(-40.05,54.53) | -0.03(-0.09,0.03) |
| Libya | 2.12(0.94,4.37) | 0.09(0.04,0.19) |  | 2.06(0.95,4.34) | 0.10(0.05,0.21) |  | -2.46(-35.72,43.01) | 7.86(-28.92,58.14) | 0.17(0.12,0.23) |
| Lithuania | 1.59(0.68,3.29) | 0.14(0.06,0.30) |  | 0.77(0.33,1.56) | 0.14(0.06,0.29) |  | -51.21(-70.05,-20.28) | 0.86(-38.08,64.81) | 0.34(0.21,0.48) |
| Luxembourg | 0.12(0.05,0.26) | 0.14(0.06,0.29) |  | 0.19(0.08,0.40) | 0.14(0.06,0.29) |  | 58.21(-7.48,163.11) | 3.46(-39.50,72.07) | 0.28(0.20,0.36) |
| Madagascar | 7.70(3.13,15.34) | 0.11(0.05,0.23) |  | 16.63(7.28,34.65) | 0.11(0.05,0.23) |  | 115.94(38.39,237.50) | -2.81(-37.71,51.90) | -0.13(-0.16,-0.09) |
| Malawi | 7.02(3.05,13.68) | 0.13(0.05,0.25) |  | 13.01(5.83,26.77) | 0.12(0.06,0.25) |  | 85.44(21.81,194.07) | -2.41(-35.90,54.75) | -0.22(-0.28,-0.17) |
| Malaysia | 7.27(3.28,14.21) | 0.09(0.04,0.17) |  | 8.94(4.16,17.68) | 0.09(0.04,0.17) |  | 23.04(-17.26,86.50) | 0.09(-32.69,51.72) | 0.04(-0.00,0.08) |
| Maldives | 0.10(0.05,0.20) | 0.08(0.04,0.15) |  | 0.10(0.04,0.19) | 0.07(0.03,0.14) |  | -0.56(-29.98,50.08) | -2.06(-31.03,47.82) | 0.07(-0.03,0.17) |
| Mali | 5.48(2.31,11.16) | 0.11(0.05,0.23) |  | 15.26(7.25,31.73) | 0.11(0.05,0.22) |  | 178.78(77.62,331.89) | -3.13(-38.28,50.08) | -0.13(-0.16,-0.09) |
| Malta | 0.15(0.07,0.30) | 0.13(0.06,0.26) |  | 0.11(0.05,0.23) | 0.13(0.06,0.28) |  | -26.04(-54.11,18.21) | 1.53(-37.01,62.27) | 0.14(0.05,0.23) |
| Marshall Islands | 0.03(0.01,0.06) | 0.10(0.04,0.21) |  | 0.02(0.01,0.05) | 0.11(0.05,0.22) |  | -10.83(-44.65,42.14) | 3.51(-35.75,65.00) | 0.06(-0.00,0.12) |
| Mauritania | 1.18(0.53,2.28) | 0.10(0.05,0.20) |  | 2.28(1.07,4.75) | 0.10(0.05,0.20) |  | 94.23(31.38,196.30) | -5.56(-36.12,44.06) | -0.22(-0.26,-0.18) |
| Mauritius | 0.37(0.17,0.77) | 0.09(0.04,0.18) |  | 0.27(0.11,0.53) | 0.09(0.04,0.18) |  | -27.32(-52.07,7.27) | 5.46(-30.45,55.65) | 0.19(0.13,0.25) |
| Mexico | 34.04(16.84,66.30) | 0.08(0.04,0.15) |  | 32.62(16.20,65.19) | 0.08(0.04,0.15) |  | -4.15(-12.35,6.96) | -3.67(-11.91,7.50) | -0.10(-0.14,-0.07) |
| Micronesia (Federated States of) | 0.06(0.03,0.13) | 0.11(0.05,0.23) |  | 0.05(0.02,0.09) | 0.11(0.05,0.22) |  | -27.95(-56.00,17.44) | -0.04(-38.96,62.93) | 0.01(-0.03,0.05) |
| Monaco | 0.01(0.00,0.01) | 0.14(0.06,0.30) |  | 0.01(0.00,0.02) | 0.14(0.06,0.28) |  | 39.88(-18.52,132.68) | 0.10(-41.69,66.51) | 0.16(0.10,0.23) |
| Mongolia | 1.60(0.71,3.24) | 0.14(0.06,0.29) |  | 1.77(0.80,3.45) | 0.14(0.06,0.26) |  | 10.92(-28.93,70.71) | -4.09(-38.54,47.62) | 0.07(-0.04,0.17) |
| Montenegro | 0.17(0.08,0.38) | 0.08(0.04,0.18) |  | 0.14(0.06,0.28) | 0.09(0.04,0.19) |  | -20.44(-45.43,18.46) | 13.48(-22.16,68.97) | 0.26(0.18,0.35) |
| Morocco | 12.70(5.76,24.69) | 0.10(0.05,0.20) |  | 13.06(5.89,27.06) | 0.10(0.05,0.21) |  | 2.83(-30.01,48.04) | -0.64(-32.37,43.04) | -0.05(-0.09,-0.01) |
| Mozambique | 9.26(4.31,18.64) | 0.12(0.06,0.25) |  | 21.17(9.80,42.07) | 0.12(0.06,0.24) |  | 128.51(51.08,249.98) | -2.56(-35.58,49.24) | -0.11(-0.15,-0.07) |
| Myanmar | 19.07(8.56,36.59) | 0.10(0.04,0.19) |  | 20.18(8.67,40.25) | 0.10(0.04,0.19) |  | 5.82(-30.46,59.70) | -2.62(-36.01,46.95) | -0.12(-0.17,-0.08) |
| Namibia | 0.93(0.42,1.96) | 0.12(0.06,0.26) |  | 1.27(0.57,2.54) | 0.12(0.05,0.24) |  | 36.22(-10.25,110.28) | -3.16(-36.19,49.50) | -0.10(-0.13,-0.07) |
| Nauru | 0.01(0.00,0.01) | 0.11(0.05,0.22) |  | 0.01(0.00,0.01) | 0.11(0.05,0.21) |  | -1.76(-40.43,56.66) | -0.20(-39.49,59.14) | -0.02(-0.10,0.07) |
| Nepal | 11.05(4.71,23.65) | 0.11(0.05,0.23) |  | 12.62(5.95,25.42) | 0.10(0.05,0.20) |  | 14.13(-24.47,75.54) | -5.16(-37.24,45.86) | -0.18(-0.25,-0.12) |
| Netherlands | 7.75(3.60,14.66) | 0.20(0.09,0.38) |  | 7.62(3.27,15.41) | 0.21(0.09,0.42) |  | -1.61(-36.68,59.36) | 2.17(-34.25,65.49) | 0.20(0.15,0.26) |
| New Zealand | 1.48(0.69,3.04) | 0.13(0.06,0.28) |  | 1.80(0.82,3.47) | 0.14(0.06,0.27) |  | 21.67(-12.77,71.74) | 2.78(-26.31,45.07) | 0.23(0.18,0.28) |
| Nicaragua | 1.53(0.72,3.15) | 0.07(0.03,0.14) |  | 1.80(0.82,3.67) | 0.07(0.03,0.14) |  | 17.16(-14.80,73.32) | 1.43(-26.24,50.05) | 0.10(0.05,0.14) |
| Niger | 5.36(2.43,11.88) | 0.11(0.05,0.24) |  | 16.80(7.27,33.84) | 0.11(0.05,0.22) |  | 213.50(94.47,380.26) | -2.42(-39.47,49.49) | -0.14(-0.19,-0.09) |
| Nigeria | 51.93(25.31,100.32) | 0.11(0.05,0.21) |  | 136.29(68.46,257.60) | 0.11(0.05,0.20) |  | 162.44(141.77,186.86) | -0.35(-8.19,8.93) | -0.07(-0.15,0.01) |
| Niue | 0.00(0.00,0.00) | 0.10(0.04,0.22) |  | 0.00(0.00,0.00) | 0.10(0.04,0.20) |  | -47.16(-64.80,-19.87) | 2.30(-31.85,55.14) | 0.07(-0.00,0.14) |
| North Macedonia | 0.57(0.25,1.19) | 0.08(0.04,0.17) |  | 0.40(0.18,0.81) | 0.09(0.04,0.18) |  | -29.47(-52.07,2.32) | 10.08(-25.20,59.70) | 0.25(0.19,0.30) |
| Northern Mariana Islands | 0.01(0.01,0.03) | 0.08(0.04,0.17) |  | 0.01(0.01,0.03) | 0.09(0.04,0.18) |  | -3.29(-32.23,44.79) | 4.58(-26.72,56.57) | 0.56(0.41,0.72) |
| Norway | 1.30(0.63,2.74) | 0.12(0.06,0.25) |  | 1.42(0.68,2.97) | 0.11(0.05,0.24) |  | 8.75(-9.22,29.38) | -2.54(-18.65,15.95) | 0.05(-0.02,0.12) |
| Oman | 0.98(0.45,1.85) | 0.10(0.04,0.18) |  | 1.32(0.58,2.61) | 0.09(0.04,0.17) |  | 34.45(-6.65,97.27) | -9.67(-37.28,32.54) | -0.18(-0.31,-0.06) |
| Pakistan | 71.20(32.90,141.11) | 0.12(0.05,0.23) |  | 124.93(60.31,259.29) | 0.11(0.06,0.24) |  | 75.46(33.88,127.70) | -2.07(-25.28,27.09) | -0.10(-0.13,-0.08) |
| Palau | 0.01(0.00,0.01) | 0.10(0.04,0.21) |  | 0.00(0.00,0.01) | 0.10(0.05,0.20) |  | -28.73(-54.13,6.23) | -0.77(-36.14,47.90) | 0.11(0.06,0.17) |
| Palestine | 1.09(0.47,2.37) | 0.09(0.04,0.20) |  | 2.06(0.91,4.17) | 0.09(0.04,0.17) |  | 90.17(30.04,177.37) | -5.73(-35.54,37.50) | -0.16(-0.21,-0.11) |
| Panama | 0.74(0.33,1.50) | 0.07(0.03,0.14) |  | 1.01(0.44,2.07) | 0.07(0.03,0.14) |  | 36.42(-5.10,99.71) | -1.61(-31.55,44.03) | -0.09(-0.12,-0.05) |
| Papua New Guinea | 2.40(1.03,4.83) | 0.11(0.05,0.23) |  | 5.63(2.43,11.52) | 0.11(0.05,0.24) |  | 134.91(47.86,265.57) | 2.07(-35.75,58.84) | 0.12(0.09,0.15) |
| Paraguay | 1.11(0.51,2.28) | 0.05(0.02,0.11) |  | 1.50(0.65,3.42) | 0.06(0.02,0.13) |  | 35.96(-13.21,96.64) | 5.34(-32.76,52.35) | 0.21(0.16,0.25) |
| Peru | 9.60(4.27,20.05) | 0.09(0.04,0.19) |  | 10.53(4.96,20.81) | 0.08(0.04,0.17) |  | 9.70(-26.13,60.86) | -6.62(-37.12,36.93) | -0.17(-0.21,-0.14) |
| Philippines | 30.11(15.06,57.43) | 0.09(0.05,0.18) |  | 42.53(21.33,81.76) | 0.09(0.05,0.18) |  | 41.26(31.32,53.32) | 1.12(-6.00,9.75) | 0.10(0.07,0.12) |
| Poland | 7.57(3.62,15.10) | 0.06(0.03,0.12) |  | 5.00(2.43,9.95) | 0.06(0.03,0.13) |  | -33.99(-42.44,-23.94) | 6.75(-6.91,23.00) | 0.14(0.05,0.22) |
| Portugal | 6.16(2.55,13.07) | 0.21(0.09,0.44) |  | 4.20(1.74,8.74) | 0.22(0.09,0.46) |  | -31.79(-57.76,16.83) | 6.09(-34.30,81.70) | 0.05(-0.05,0.14) |
| Puerto Rico | 0.98(0.44,2.02) | 0.07(0.03,0.15) |  | 0.50(0.21,1.02) | 0.08(0.03,0.16) |  | -49.26(-67.85,-22.14) | 3.44(-34.47,58.71) | 0.05(0.01,0.09) |
| Qatar | 0.14(0.06,0.28) | 0.09(0.04,0.18) |  | 0.48(0.24,0.91) | 0.08(0.04,0.15) |  | 248.03(159.37,394.16) | -10.77(-33.50,26.69) | -0.23(-0.33,-0.13) |
| Republic of Korea | 12.03(5.29,25.48) | 0.08(0.03,0.16) |  | 6.11(2.73,12.76) | 0.07(0.03,0.15) |  | -49.24(-67.09,-23.55) | -3.56(-37.48,45.24) | -0.05(-0.21,0.12) |
| Republic of Moldova | 2.18(0.92,4.49) | 0.14(0.06,0.28) |  | 1.02(0.45,2.11) | 0.15(0.06,0.30) |  | -53.21(-71.63,-25.36) | 6.27(-35.57,69.51) | 0.29(0.14,0.43) |
| Romania | 6.80(2.84,13.69) | 0.09(0.04,0.18) |  | 3.80(1.71,7.63) | 0.09(0.04,0.19) |  | -44.10(-60.89,-17.67) | 3.79(-27.38,52.87) | -0.07(-0.18,0.04) |
| Russian Federation | 70.89(35.48,139.23) | 0.16(0.08,0.31) |  | 52.45(25.71,105.85) | 0.16(0.08,0.31) |  | -26.01(-33.56,-18.30) | -1.06(-11.15,9.25) | -0.05(-0.23,0.13) |
| Rwanda | 5.02(2.08,9.74) | 0.12(0.05,0.24) |  | 7.53(3.37,15.13) | 0.12(0.05,0.24) |  | 49.95(0.49,135.12) | -3.61(-35.40,51.15) | -0.12(-0.22,-0.03) |
| Saint Kitts and Nevis | 0.01(0.01,0.03) | 0.08(0.04,0.15) |  | 0.01(0.01,0.02) | 0.08(0.04,0.17) |  | -23.29(-48.94,12.05) | 0.22(-33.29,46.38) | 0.08(0.03,0.13) |
| Saint Lucia | 0.05(0.02,0.10) | 0.07(0.03,0.15) |  | 0.03(0.01,0.07) | 0.08(0.04,0.16) |  | -31.66(-54.06,4.24) | 8.76(-26.89,65.90) | 0.35(0.29,0.41) |
| Saint Vincent and the Grenadines | 0.04(0.02,0.08) | 0.08(0.03,0.15) |  | 0.03(0.01,0.05) | 0.08(0.03,0.16) |  | -36.20(-56.40,-8.33) | 1.75(-30.47,46.19) | 0.10(0.05,0.14) |
| Samoa | 0.09(0.04,0.19) | 0.10(0.04,0.21) |  | 0.09(0.04,0.18) | 0.09(0.04,0.18) |  | 2.66(-31.06,52.42) | -6.84(-37.44,38.31) | -0.18(-0.20,-0.15) |
| San Marino | 0.01(0.00,0.02) | 0.13(0.06,0.28) |  | 0.01(0.00,0.02) | 0.14(0.06,0.28) |  | 10.58(-29.79,72.09) | 6.03(-32.68,65.01) | 0.24(0.11,0.36) |
| Sao Tome and Principe | 0.07(0.03,0.14) | 0.10(0.05,0.20) |  | 0.10(0.04,0.20) | 0.10(0.04,0.20) |  | 43.35(-4.23,106.30) | -1.60(-34.25,41.61) | -0.14(-0.17,-0.10) |
| Saudi Arabia | 8.64(3.89,17.26) | 0.11(0.05,0.21) |  | 10.71(4.83,22.30) | 0.11(0.05,0.22) |  | 24.04(-13.95,86.11) | 0.51(-30.27,50.81) | 0.13(0.07,0.19) |
| Senegal | 4.72(1.93,9.74) | 0.11(0.04,0.22) |  | 8.93(3.85,19.13) | 0.11(0.05,0.24) |  | 89.14(23.33,188.62) | 3.65(-32.42,58.16) | 0.10(0.07,0.13) |
| Serbia | 2.42(1.13,5.07) | 0.08(0.04,0.17) |  | 1.79(0.76,3.61) | 0.10(0.04,0.19) |  | -25.93(-50.63,12.18) | 14.67(-23.56,73.68) | 0.21(0.13,0.28) |
| Seychelles | 0.03(0.01,0.06) | 0.09(0.04,0.19) |  | 0.03(0.01,0.05) | 0.09(0.04,0.18) |  | -3.89(-33.89,42.02) | -1.64(-32.35,45.35) | -0.05(-0.13,0.02) |
| Sierra Leone | 2.52(1.07,4.95) | 0.11(0.05,0.22) |  | 5.03(2.26,10.48) | 0.11(0.05,0.23) |  | 99.54(22.97,228.79) | -3.28(-40.40,59.37) | -0.02(-0.07,0.02) |
| Singapore | 0.69(0.30,1.39) | 0.08(0.03,0.15) |  | 0.69(0.31,1.42) | 0.07(0.03,0.14) |  | -0.67(-33.44,50.04) | -12.33(-41.25,32.44) | -0.36(-0.55,-0.17) |
| Slovakia | 1.50(0.69,3.19) | 0.09(0.04,0.18) |  | 1.00(0.46,2.06) | 0.09(0.04,0.18) |  | -32.98(-54.04,-1.32) | 4.93(-28.05,54.50) | 0.08(-0.03,0.18) |
| Slovenia | 0.44(0.21,0.85) | 0.08(0.04,0.15) |  | 0.34(0.15,0.67) | 0.08(0.04,0.17) |  | -24.15(-48.01,11.16) | 4.33(-28.50,52.88) | -0.15(-0.27,-0.03) |
| Solomon Islands | 0.17(0.08,0.34) | 0.09(0.04,0.17) |  | 0.29(0.14,0.59) | 0.09(0.04,0.18) |  | 68.36(11.98,148.75) | -0.93(-34.11,46.37) | 0.01(-0.02,0.03) |
| Somalia | 6.51(2.90,13.25) | 0.14(0.06,0.28) |  | 17.64(7.46,34.92) | 0.14(0.06,0.27) |  | 170.93(71.01,288.01) | 0.29(-36.70,43.63) | -0.08(-0.13,-0.02) |
| South Africa | 21.69(10.70,41.77) | 0.12(0.06,0.24) |  | 24.45(11.56,47.87) | 0.12(0.06,0.24) |  | 12.70(-7.07,33.20) | -0.29(-17.78,17.84) | -0.02(-0.09,0.05) |
| South Sudan | 3.96(1.89,7.81) | 0.12(0.06,0.24) |  | 6.58(2.64,14.16) | 0.12(0.05,0.26) |  | 66.12(5.96,157.09) | -0.52(-36.55,53.96) | -0.07(-0.12,-0.02) |
| Spain | 9.00(4.13,18.73) | 0.08(0.04,0.17) |  | 8.12(3.73,17.17) | 0.09(0.04,0.19) |  | -9.71(-38.71,33.37) | 14.28(-22.43,68.80) | 1.05(0.87,1.23) |
| Sri Lanka | 6.41(2.84,12.92) | 0.09(0.04,0.18) |  | 5.83(2.45,12.15) | 0.08(0.04,0.18) |  | -9.03(-37.89,36.85) | -4.55(-34.83,43.60) | -0.25(-0.28,-0.21) |
| Sudan | 12.11(5.28,24.69) | 0.11(0.05,0.22) |  | 22.12(9.95,44.27) | 0.10(0.05,0.21) |  | 82.77(27.23,174.73) | -5.55(-34.25,41.98) | -0.18(-0.21,-0.15) |
| Suriname | 0.14(0.06,0.28) | 0.08(0.03,0.17) |  | 0.15(0.07,0.32) | 0.08(0.03,0.17) |  | 10.86(-22.32,65.59) | -0.16(-30.04,49.14) | 0.04(0.01,0.08) |
| Sweden | 2.07(0.93,4.22) | 0.10(0.04,0.20) |  | 2.32(1.13,4.82) | 0.10(0.05,0.20) |  | 12.22(-15.86,52.19) | -2.10(-26.60,32.77) | -0.18(-0.48,0.12) |
| Switzerland | 2.03(0.91,4.22) | 0.13(0.06,0.27) |  | 2.36(1.03,4.88) | 0.13(0.06,0.28) |  | 16.45(-28.97,81.42) | 4.20(-36.44,62.34) | 0.28(0.22,0.35) |
| Syrian Arab Republic | 6.93(3.18,13.66) | 0.09(0.04,0.19) |  | 5.18(2.16,10.25) | 0.10(0.04,0.19) |  | -25.18(-53.57,8.23) | 1.12(-37.25,46.27) | -0.16(-0.25,-0.07) |
| Taiwan (Province of China) | 6.13(2.86,12.15) | 0.08(0.04,0.17) |  | 3.23(1.46,6.50) | 0.08(0.04,0.16) |  | -47.32(-64.96,-23.72) | -4.82(-36.70,37.82) | -0.15(-0.23,-0.07) |
| Tajikistan | 3.40(1.53,6.52) | 0.12(0.05,0.23) |  | 5.63(2.53,11.48) | 0.13(0.06,0.26) |  | 65.29(8.41,155.92) | 6.65(-30.05,65.12) | 0.37(0.27,0.47) |
| Thailand | 20.55(9.17,39.99) | 0.09(0.04,0.18) |  | 11.92(5.17,24.00) | 0.09(0.04,0.18) |  | -42.00(-62.17,-11.05) | -3.34(-36.96,48.25) | -0.14(-0.18,-0.09) |
| Timor-Leste | 0.39(0.19,0.80) | 0.10(0.05,0.20) |  | 0.63(0.28,1.34) | 0.09(0.04,0.19) |  | 60.07(5.85,137.92) | -5.03(-37.20,41.16) | -0.17(-0.22,-0.11) |
| Togo | 2.45(1.05,5.02) | 0.11(0.05,0.23) |  | 4.75(2.24,9.65) | 0.11(0.05,0.23) |  | 93.69(23.74,205.33) | -0.14(-36.20,57.42) | 0.02(-0.02,0.06) |
| Tokelau | 0.00(0.00,0.00) | 0.09(0.04,0.18) |  | 0.00(0.00,0.00) | 0.09(0.04,0.19) |  | -33.38(-57.01,-1.89) | -1.23(-36.27,45.45) | 0.09(0.00,0.18) |
| Tonga | 0.05(0.02,0.10) | 0.09(0.04,0.18) |  | 0.04(0.02,0.09) | 0.09(0.04,0.18) |  | -7.66(-39.35,35.79) | -0.63(-34.73,46.14) | -0.04(-0.06,-0.02) |
| Trinidad and Tobago | 0.40(0.18,0.85) | 0.08(0.04,0.16) |  | 0.28(0.12,0.57) | 0.08(0.03,0.16) |  | -28.17(-50.70,0.21) | 2.52(-29.64,43.02) | -0.05(-0.17,0.07) |
| Tunisia | 3.57(1.59,7.15) | 0.09(0.04,0.18) |  | 3.16(1.42,6.53) | 0.09(0.04,0.18) |  | -11.47(-39.22,29.81) | -1.48(-32.37,44.45) | -0.07(-0.15,0.02) |
| Turkey | 24.05(11.12,51.41) | 0.09(0.04,0.19) |  | 20.91(9.43,40.49) | 0.08(0.04,0.16) |  | -13.07(-40.91,28.80) | -6.14(-36.20,39.06) | -0.28(-0.33,-0.23) |
| Turkmenistan | 2.32(1.04,4.88) | 0.12(0.06,0.26) |  | 2.53(1.13,5.11) | 0.13(0.06,0.26) |  | 9.01(-30.69,64.70) | 4.82(-33.35,58.37) | 0.21(0.13,0.29) |
| Tuvalu | 0.00(0.00,0.01) | 0.11(0.05,0.23) |  | 0.01(0.00,0.01) | 0.11(0.05,0.21) |  | 11.26(-24.51,79.16) | -3.22(-34.34,55.83) | -0.01(-0.06,0.04) |
| Uganda | 12.54(5.48,25.71) | 0.12(0.05,0.25) |  | 29.85(13.65,61.23) | 0.12(0.05,0.25) |  | 138.07(49.34,267.43) | -1.72(-38.34,51.69) | -0.04(-0.07,-0.01) |
| Ukraine | 23.24(9.88,48.83) | 0.15(0.07,0.32) |  | 13.92(5.87,27.54) | 0.16(0.07,0.33) |  | -40.13(-63.94,-0.81) | 6.70(-35.74,76.79) | 0.12(-0.04,0.27) |
| United Arab Emirates | 0.71(0.33,1.40) | 0.10(0.05,0.20) |  | 1.73(0.83,3.58) | 0.10(0.05,0.21) |  | 145.77(72.83,261.58) | 2.51(-27.91,50.82) | -0.08(-0.29,0.14) |
| United Kingdom | 18.48(9.20,39.01) | 0.12(0.06,0.26) |  | 19.77(9.75,39.18) | 0.13(0.06,0.25) |  | 6.98(-1.50,16.23) | 1.37(-6.66,10.14) | 0.24(0.19,0.30) |
| United Republic of Tanzania | 16.80(7.83,33.30) | 0.11(0.05,0.22) |  | 33.99(14.60,71.03) | 0.11(0.05,0.23) |  | 102.34(28.40,202.93) | -2.15(-37.90,46.50) | -0.09(-0.13,-0.06) |
| United States of America | 87.26(42.72,173.75) | 0.12(0.06,0.23) |  | 95.73(45.25,190.58) | 0.12(0.06,0.23) |  | 9.71(-0.25,20.77) | -0.09(-9.16,9.98) | -0.12(-0.21,-0.03) |
| United States Virgin Islands | 0.03(0.01,0.07) | 0.08(0.03,0.16) |  | 0.01(0.01,0.03) | 0.08(0.04,0.16) |  | -55.57(-68.79,-35.33) | 1.73(-28.54,48.07) | 0.13(0.07,0.19) |
| Uruguay | 0.51(0.23,1.03) | 0.05(0.02,0.10) |  | 0.44(0.19,0.93) | 0.05(0.02,0.10) |  | -13.10(-45.65,41.04) | 3.60(-35.20,68.16) | -0.07(-0.28,0.13) |
| Uzbekistan | 12.66(5.47,25.59) | 0.12(0.05,0.24) |  | 15.70(7.37,30.39) | 0.12(0.06,0.24) |  | 23.98(-19.29,90.83) | 4.97(-31.67,61.56) | 0.29(0.20,0.39) |
| Vanuatu | 0.09(0.04,0.18) | 0.11(0.05,0.22) |  | 0.15(0.07,0.30) | 0.10(0.04,0.21) |  | 74.19(14.73,185.89) | -1.41(-35.07,61.81) | -0.02(-0.06,0.02) |
| Venezuela (Bolivarian Republic of) | 7.16(3.30,14.50) | 0.08(0.04,0.16) |  | 6.68(3.07,12.76) | 0.08(0.04,0.15) |  | -6.60(-33.99,38.97) | -3.14(-31.55,44.12) | -0.06(-0.12,-0.01) |
| Viet Nam | 28.44(13.27,56.23) | 0.08(0.04,0.17) |  | 25.58(12.11,50.00) | 0.08(0.04,0.16) |  | -10.04(-37.05,30.74) | -4.21(-32.97,39.21) | -0.05(-0.17,0.06) |
| Yemen | 9.30(4.06,18.84) | 0.11(0.05,0.22) |  | 18.53(7.76,39.65) | 0.11(0.04,0.23) |  | 99.35(23.32,215.60) | -4.07(-40.65,51.87) | -0.25(-0.30,-0.19) |
| Zambia | 5.84(2.78,11.45) | 0.12(0.06,0.24) |  | 12.54(5.68,24.12) | 0.12(0.05,0.23) |  | 114.64(37.85,227.54) | -3.49(-38.02,47.27) | -0.18(-0.22,-0.15) |
| Zimbabwe | 7.26(3.37,14.50) | 0.12(0.06,0.24) |  | 10.11(4.53,19.37) | 0.13(0.06,0.24) |  | 39.37(-3.44,105.17) | 5.12(-27.16,54.76) | 0.24(0.19,0.29) |
